# Demonstrating the value of beaches for adaptation to future coastal flood risk

**DOI:** 10.1038/s41467-023-39168-z

**Published:** 2023-06-12

**Authors:** Alexandra Toimil, Iñigo J. Losada, Moisés Álvarez-Cuesta, Gonéri Le Cozannet

**Affiliations:** 1 IHCantabria - Instituto de Hidráulica Ambiental de la Universidad de Cantabria, Isabel Torres 15, 39011 Santander, Spain; 2 Bureau de Recherches Géologiques et Minières “BRGM”, French Geological Survey, 3 Avenue Claude Guillemin, CEDEX, 45060 Orléans, France

**Keywords:** Climate-change impacts, Natural hazards, Climate change

## Abstract

Cost-effective coastal flood adaptation requires a realistic valuation of losses, costs and benefits considering the uncertainty of future flood projections and limited resources for adaptation. Here we present an approach to quantify the flood protection benefits of beaches accounting for the dynamic interaction of storm erosion, long-term shoreline evolution and flooding. We apply the method in Narrabeen-Collaroy (Australia) considering uncertainty in different shared socioeconomic pathways, sea-level rise projections, and beach conditions. By 2100, results show that failing to consider erosion can underestimate flood damage by a factor of 2 and maintaining present-day beach width can avoid 785 million AUD worth assets from flood damage. By 2050, the flood protection and recreational benefits of holding the current mean shoreline could be more than 150 times the cost of nourishment. Our results give insight on the benefits of beaches for adaptation and can help accelerate financial instruments for restoration.

## Abstract (web summary)

This paper presents a method for quantifying the benefits of beaches in reducing storm and long-term coastal flood risk. This method can contribute to cost-effective decision-making on climate change adaptation in many of the world’s coasts.

## Introduction

The future impacts of climate change and of mean sea-level rise (SLR) are expected to have a profound effect on the coastal zone^1,2^, although the magnitude of these impacts is still uncertain^3,4^. Higher water levels will lead to the permanent inundation of some low-lying coastal zones^5^ and more frequent extreme flood events^6–8^, which will in turn modify coastal landscapes increasing the exposure of coastal communities and assets^9,10^. Essential features of coastal landscapes include ecosystems such as wetlands, mangroves, coral reefs, and beach and dune systems, which provide multiple benefits that have begun to decline and will continue to do so at rapid rates without climate change mitigation, risk management and adaptation ^11–14^. One of the coastal ecosystem services under threat is flood protection, which is particularly important along densely populated and developed coastlines^15,16^.

The flood protection services provided by mangroves and coral reefs have been economically assessed in the literature considering climate and degradation scenarios^17–19^. This has contributed to the benefits of conserving and restoring them being increasingly recognised by scientists, multilateral and governmental agencies, and the insurance industry^20,21^. As for beaches, there are several studies that analyse their effectiveness as natural flood defences^22–25^, however, additional work is needed to understand their benefits in flood risk reduction. Knowing this information can be of key importance as beaches represent one-third of the world’s coasts^26^, they are subject to the action of storms and rising mean sea levels^27,28^, and their maintenance has long been under discussion^29,30^. Cost-effective decision-making on beach conservation would benefit from assessing the trade-off between the cost of an action and the benefit, including the economic benefit, that would accrue from its implementation.

Previous efforts to monetise beach services have examined the loss of human recreation due to erosion^31–33^, the influence of erosion (and beach nourishment policies) on coastal property values^34–37^, the tax rates needed to fund adaptation projects^38,39^, and the willingness-to-pay for erosion prevention^40–42^ and flood protection ^43–45^. Here, our goal is to advance knowledge on how to quantify the flood protection value of beaches to deliver relevant information for adaptation decisions.

We propose a dynamic approach based on the avoided damage cost method, which is similar to that applied for mangroves and coral reefs (e.g., refs. ^17,18^), but considers the specificities of beaches. We acknowledge that coastal flood protection is strongly dependent on the shoreline response to coastal dynamics. Therefore, our approach accounts for the dynamic interaction of shoreline evolution and flooding by coupling both processes at different time scales. This allows us to evaluate how the shoreline evolves under extreme events and due to long-term changes and how this evolution affects the total water level (TWL) and the propagation of flooding inland, which are in turn influenced by shoreface geometry and terrain heights. We also assume that robust adaptation requires not only a good understanding of physical processes but also sufficient uncertainty sampling to accommodate decisions in different contexts and time horizons and for different levels of risk and uncertainty tolerance.

**Fig. 1:**
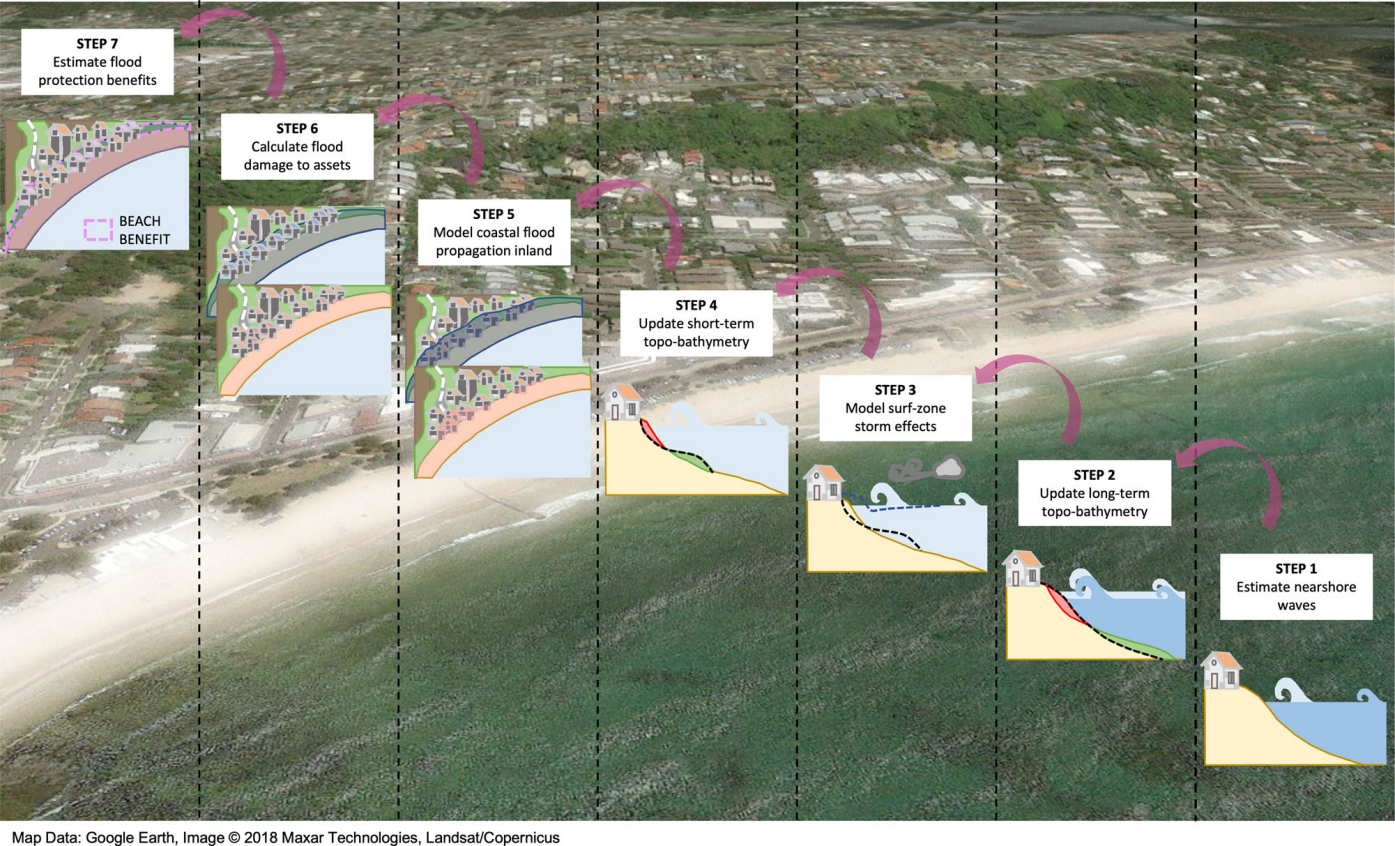
Key steps for estimating the flood protection services provided by beaches. From right to left. Step 1: Nearshore wave downscaling. Step 2: Integration of erosion on topo-bathymetries due to sea-level rise. Step 3: Modelling of surf-zone morphodynamics and hydrodynamics. Step 4: Integration of storm erosion on long-term topo-bathymetries. Step 5: Modelling of coastal flood propagation inland. Step 6: Calculation of flood damage to assets. Step 7: Calculation of the flood protection benefits of beaches in terms of avoided flood damage.

We use the widely studied Narrabeen-Collaroy beach system (Australia) as an illustration to showcase our approach. The need for coupling flooding and erosion in beach protection benefits assessment is highlighted by comparing flooding results with and without including coastal erosion at the storm scale and in the long term. The flooding scenarios are built for the present-day 30-year return period storm combined with AR6 SLR^46^ in 2050 and 2100. The scenarios with erosion consider the effect of storm and SLR erosion on the TWL, flooded area, and flood damage to assets. As represented in seven steps in Fig. 1, we use process-based models to downscale offshore waves, compute storm hydro- and morphodynamics and propagate flooding inland. Throughout this process, we update the present-day topo-bathymetry to first incorporate the action of SLR and then that of the storm. We quantify flood damage by combining our flood maps with data on spatially distributed land and buildings value. Further details on the approach can be found in the “Methods” section.

We applied the dynamic approach to obtain flood damage with erosion and the traditional static approach to obtain flood damage without erosion. The scenarios without erosion assume that the coastline and beach morphology are fixed over time and that changes in flood damage are caused by changes in the TWL and subsequent flooding. We define the beach flood protected area as the increase in the flooded area due to erosion. The beach flood protected value is the flood damage that occurs in the flood protected area and can be considered as the benefit we obtain in terms of avoided flood damage if the present shoreline is maintained in the face of storms and SLR. We provide estimates of TWL (dynamic wave setup and sea-level components), flooded area, flood damage, and avoided flood damage for 2020, 2050 and 2100 considering uncertainty in the choice of emissions scenarios (shared socioeconomic pathway—radiative forcing level SSP2-4.5 and SSP5-8.5), SLR driving processes of different confidence (medium and low), and SLR trajectories (associated with percentiles P5th, P50th and P95th). In addition, we assess the trade-off between the benefits and nourishment costs of maintaining present-day mean beach width.

## Results

### The flood protection value concept

**Fig. 2:**
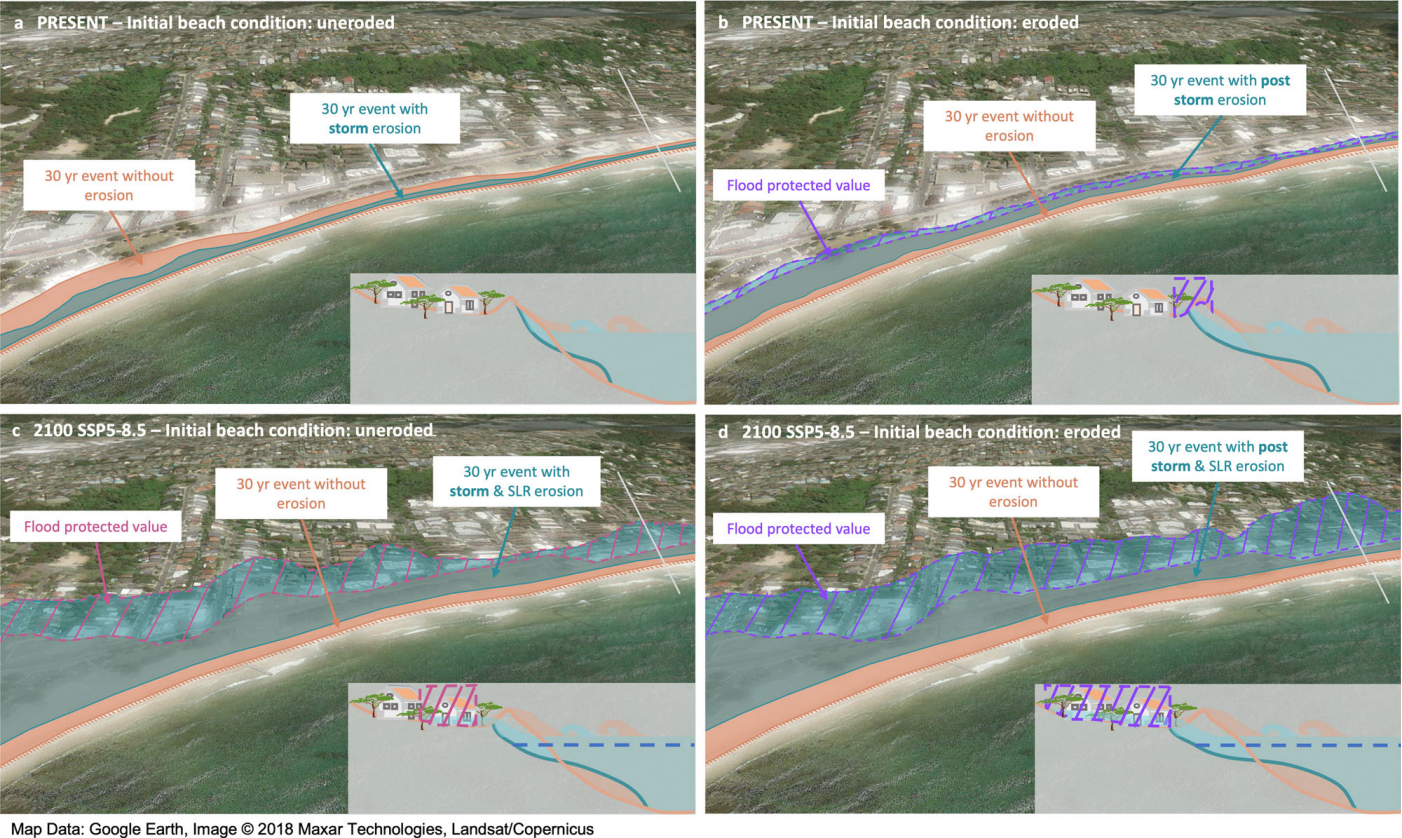
Representation of the flood protected area and value at Narrabeen-Collaroy. The flood protected area and value are the magenta and purple shaded regions, respectively, in planform and profile sketches. Turquoise and orange areas denote the flood extent from a 30-year total water level (TWL) event with and without coastal erosion, respectively. **a** Storm erosion due to the 30-year event on an uneroded beach (present). **b** Storm erosion due to the 30-year event on the beach already eroded by a recent previous storm (present). **c** Storm erosion due to the 30-year event on the beach already eroded by sea-level rise (SLR) (2100). **d** Storm erosion due to the 30-year event on the beach already eroded by sea-level rise and a recent previous storm (2100). Note that in **a** reduced TWL due to storm erosion leads to lower flooding than if no erosion is considered; and in **b** the initial erosion condition of the beach is such that regardless the reduction in TWL, flooding is higher than if no erosion is considered.

**Fig. 3:**
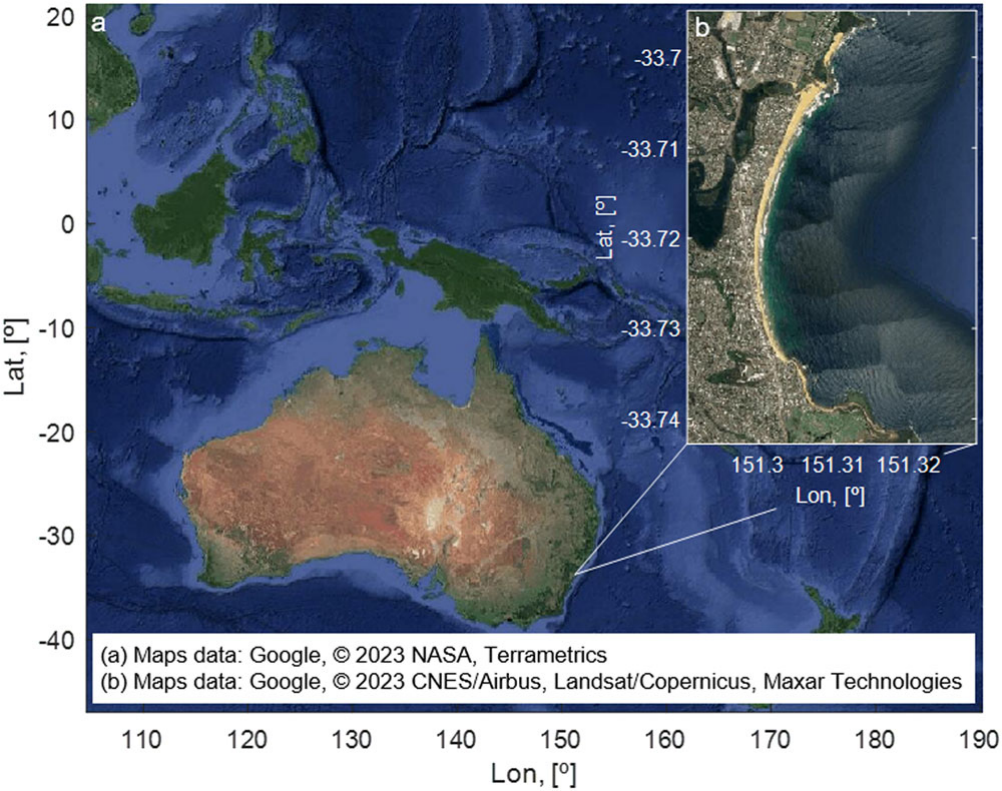
Geographic location of the Narrabeen-Collaroy beach system. Narrabeen-Collaroy is a 3.6 km long embayed beach that is located in 20 km north of Sydney (Australia). The beach system is bounded by Long Reef Point to the south and Narrabeen Headland to the north, with beachfront houses and apartments on most of its backside.

Figure 2 shows the flood protection value of Narrabeen-Collaroy (Fig. 3) for the impact of a 30-year storm now and in the future. This shows the key role played by the beach in protecting coastal assets. The interaction between storm hydrodynamics and morphodynamics results in an eroded shoreface and in an attenuated wave contribution to the TWL. At present, reduced TWL can result in lower or higher flooding than without erosion depending on whether storm erosion takes place on a beach uneroded (storm condition, Fig. 2a) or previously eroded due to a recent storm without having time to recover (poststorm condition, Fig. 2b), respectively. In the future, storm erosion compounded by SLR-driven chronic coastline retreat will further narrow the coastal landscape. Even if wave dissipation occurs, this narrowing combined with SLR-driven higher TWL will lead to greater flooding (Fig. 2c, d).

### The total water level

**Fig. 4:**
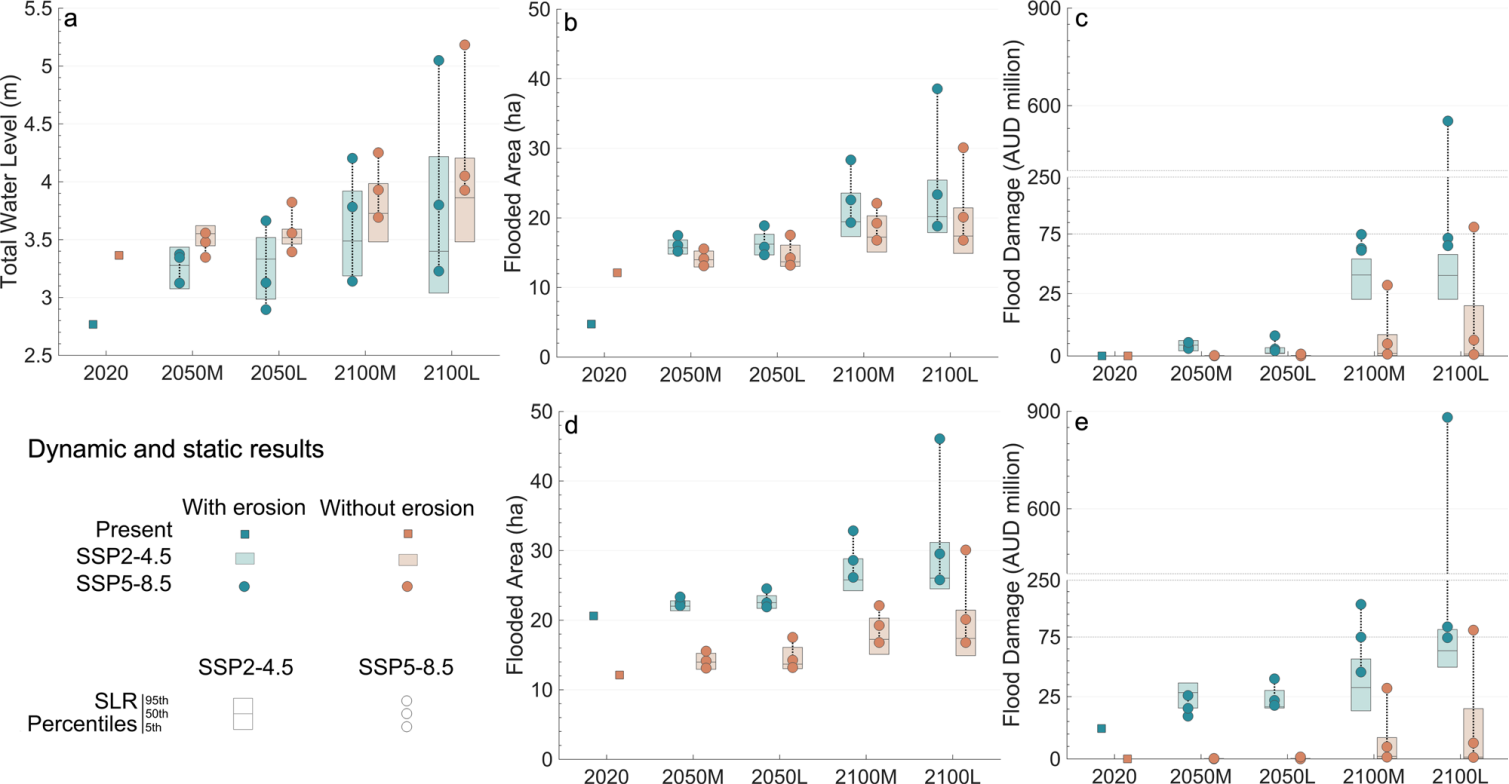
Dynamic and static results at Narrabeen-Collaroy. Results of the application of the dynamic and static approaches (turquoise and orange, respectively) are provided for 2020, 2050 and 2100, for two sea-level rise (SLR) confidence scenarios (medium and low confidence, M and L, respectively), two emissions scenarios (SSP2-4.5 and SSP5-8.5, boxes and circles, respectively) and three SLR trajectories per each confidence scenario and SSP associated with three percentiles of the distribution (5th, 50th, and 95th, horizontal lines of the boxes and circles for the SSP2-4.5 and SSP5-8.5, respectively). **a** Total water level in a profile located in the centre of the beach (m). **b** Flooded area for the beach in storm conditions (ha) measured from the 2018 mean high-water line. **c** Flood damage for the beach in storm conditions (million AUD). **d** Flooded area for the beach in poststorm conditions (ha) measured from the 2018 mean high-water line. **e** Flood damage for the beach in poststorm conditions (ha). Note that to improve visualisation, in **c** and **e** y-axis is broken and results below 75 million AUD are represented in a distant-proportional distorted scale. The plots were created using MATLAB R2022a.

Figure 4a demonstrates the influence of storm erosion and profile geometry on TWL estimates. The 30-year storm causes a redistribution of sediment in the profile (erosion in the upper part and deposition in the lower part) that reduces the foreshore slope and thus the wave setup and the TWL. For example, TWL can be reduced up to 18% in 2020 and 15–20% in 2100 (SLR P5th) compared to that in a fixed shoreline. This dissipative effect is less apparent for high SLR percentiles and low-confidence scenarios. SLR moves profiles upwards and landwards but maintains their shapes, so that any change in TWL in this regard is due to the geometric singularities of the profile swash zone based on the raised sea levels rather than by a systematic smoothing of the foreshore slope. The TWL spread range is larger and increases with time and in response to the most unfavourable SSP and the lowest SLR confidence.

### The flooded area

As illustrated in Fig. 2, when flooding starts to propagate inland during a storm event, one key factor in the flooded area is the beach condition at the time the storm hits the coastline. Figure 4b, d shows the flooded area in Narrabeen-Collaroy for storm and poststorm conditions. For storm conditions, storm erosion has much less weight than that of SLR in the flooded area. This explains why higher without-erosion TWLs lead to flooded areas that, compared with those with erosion, are larger for present (by 150%) but smaller for future scenarios (10–22%). In poststorm conditions, however, storm erosion due to the cumulative effect of two consecutive back-to-back storms gains importance. This makes the without-erosion flooded area smaller than that with erosion by 40%, an effect that can be of growing importance in the future (by up to 45%). The topography of the upper shoreface, dunes and low-lying areas is another key factor, as small changes in the TWL can lead to great variations in the flooded area.

### The flood damage

Figure 4c, e displays the results of the flood damage in million AUD. Flood damage depends on the flooded area, the coastal assets (e.g., land parcels and buildings and their cadastral and real estate market values, which are affected by land use types and the distance from the coastline), and the damage functions that characterise coastal assets vulnerability to flooding. The differences in flood damage between the scenarios with and without erosion are stronger than those for the TWL and the flooded area, especially by 2100. The assumption that the shoreline will not change in the future can lead to 60–100% lower results than those that consider the joint effect of storm and SLR erosion. For the worst-case scenario, flood damage can reach 7 or 34 million AUD in 2050 and 553 or 880 million AUD in 2100 based on the beach condition (storm or poststorm, respectively). These results highlight that the widely-used static approach that neglects the dynamic behaviour of the coastline in coastal flood modelling can give rise to misleading flood damage and compromise adaptation planning.

### Variance partition analysis

**Fig. 5:**
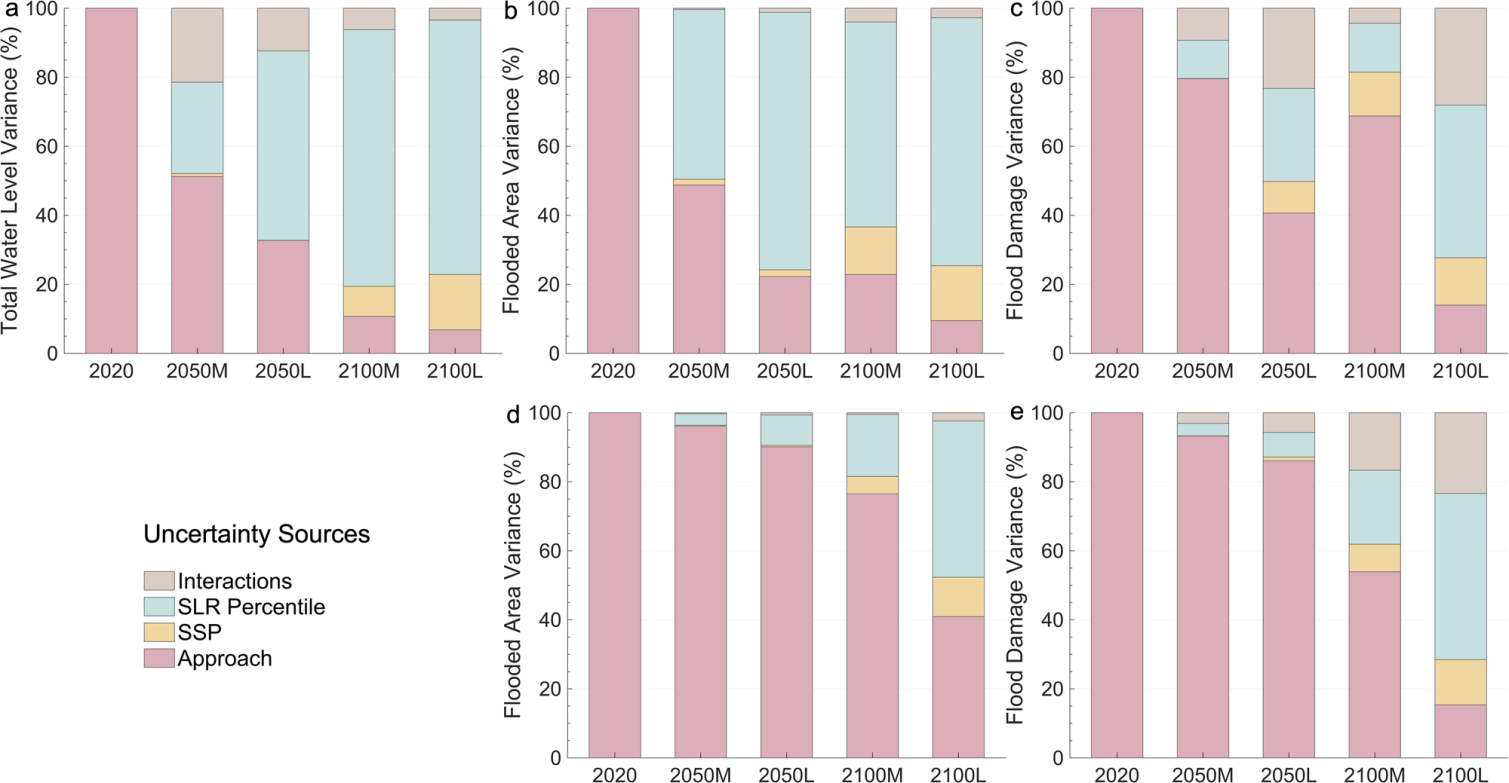
Attribution of the variance of the Narrabeen-Collaroy results to their sources of uncertainty. Break-down of uncertainty (%) in the total water level (**a**), the flooded area for the beach in storm conditions (**b**), the flood damage for the beach in storm conditions (**c**), the flooded area for the beach in poststorm conditions (**d**), and the flood damage for the beach in poststorm conditions (**e**). Variance fractions are attributed to the approach (dynamic or static), the emissions scenario denoted here for simplicity as SSP (SSP2-4.5 or SSP5-8.5), the sea-level rise (SLR) percentile (trajectory associated with the 5th, 50th or 95th percentiles), and interactions (excluding interactions between SLR components due to their dependency). Results are shown for 2020, 2050 and 2100 for two SLR confidence scenarios (medium and low confidence, M and L, respectively). The plots were created using MATLAB R2022a.

Figure 5 presents a variance partitioning analysis that disentangles the relative contributions of SSPs, SLR percentiles, and modelling approaches (dynamic and static) to the TWL, the flooded area and flood damage. The results are decomposed by time horizon and SLR confidence considering storm and poststorm beach conditions. For most future scenarios, the importance of the approach increases at each level of the modelling chain (7–51%, 9–49%, and 14–80% for the TWL, the flooded area and flood damage, respectively) and is especially large for the poststorm condition (16–91%), dominating over SLR uncertainty even in 2100. This highlights the relevance of considering the coupled effect of flooding and erosion and the actual physical characteristics (i.e., profile geometry, terrain heights and distribution of flood-damaged assets). Overall, the divergence between SLR percentiles is greater for low than medium-confidence scenarios, as is the SLR contribution to the variance in the results (7–75% against 4–59%). The relative contribution of SSPs is virtually negligible in 2050 but increases in 2100 (more than 16% and 18% for SLR medium and low confidence, respectively).

### Flood protection benefits

**Fig. 6:**
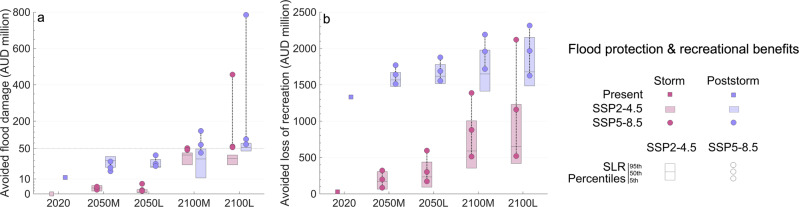
Benefits of maintaining the present-day coastline at Narrabeen-Collaroy. Results consider storm and poststorm conditions (magenta and purple, respectively). **a** Flood protection benefits in terms of avoided flood damage (million AUD). **b** Recreational benefits in terms of avoided loss of recreation (million AUD). Results are shown for 2020, 2050 and 2100 for two sea-level rise (SLR) confidence scenarios (medium and low confidence, M and L, respectively), two emissions scenarios (SSP2-4.5 and SSP5-8.5, boxes and circles, respectively) and three SLR trajectories per each confidence scenario and SSP associated with three percentiles of the distribution (5th, 50th, and 95th, horizontal lines of the boxes and circles for the SSP2-4.5 and SSP5-8.5, respectively). Note that to improve visualisation, in **a** results below 50 million AUD are represented in a distant-proportional distorted scale. The plots were created using MATLAB R2022a.

Figure 6a shows the flood protection value of Narrabeen-Collaroy in terms of avoided flood damage for the impact of a 30-year storm now and in the future. The greater the avoided damage, the greater the benefit of the beach. At present, having an eroded or non-eroded beach when a storm occurs makes a difference of 11 million AUD in avoided flood damage. In 2050, the flood protection benefit of the beach in a storm clustering situation is 42% greater than that in the face of a single storm for the SSP5-8.5 medium-confidence SLR P95th, and this difference will increase with time (up to 186% in 2100). However, in the event of a single storm, the benefit of maintaining the present-day beach width is also substantial, especially as SLR increases and SSPs diverge. Holding the present-day shoreline can result in a range of avoided flood damage of 1–7 million AUD in 2050 and 22–456 million AUD in 2100 (up to 146 and 785 million AUD, respectively, for poststorm conditions).

### Recreational benefits

In addition to flood protection, beaches can provide recreational services. Figure 6b shows the recreational benefits of Narrabeen-Collaroy in terms of avoided loss of recreation. We define the avoided loss of recreation as the recreational value associated with the loss of beach area that would result from erosion. The recreational value of beaches is non-market and reflect the welfare and happiness that people gain from using the resource. In Narrabeen-Collaroy it can range from 65–596 million AUD in 2050 and 355–2120 million AUD in 2100 (up to 1877 and 2300 million AUD, respectively, for poststorm conditions). These flood protection and recreational benefits are an upper bound since beaches can recover fully (or partially) after storms provided that the limit of their natural resilience is not exceeded.

### Benefit-cost analysis

**Fig. 7:**
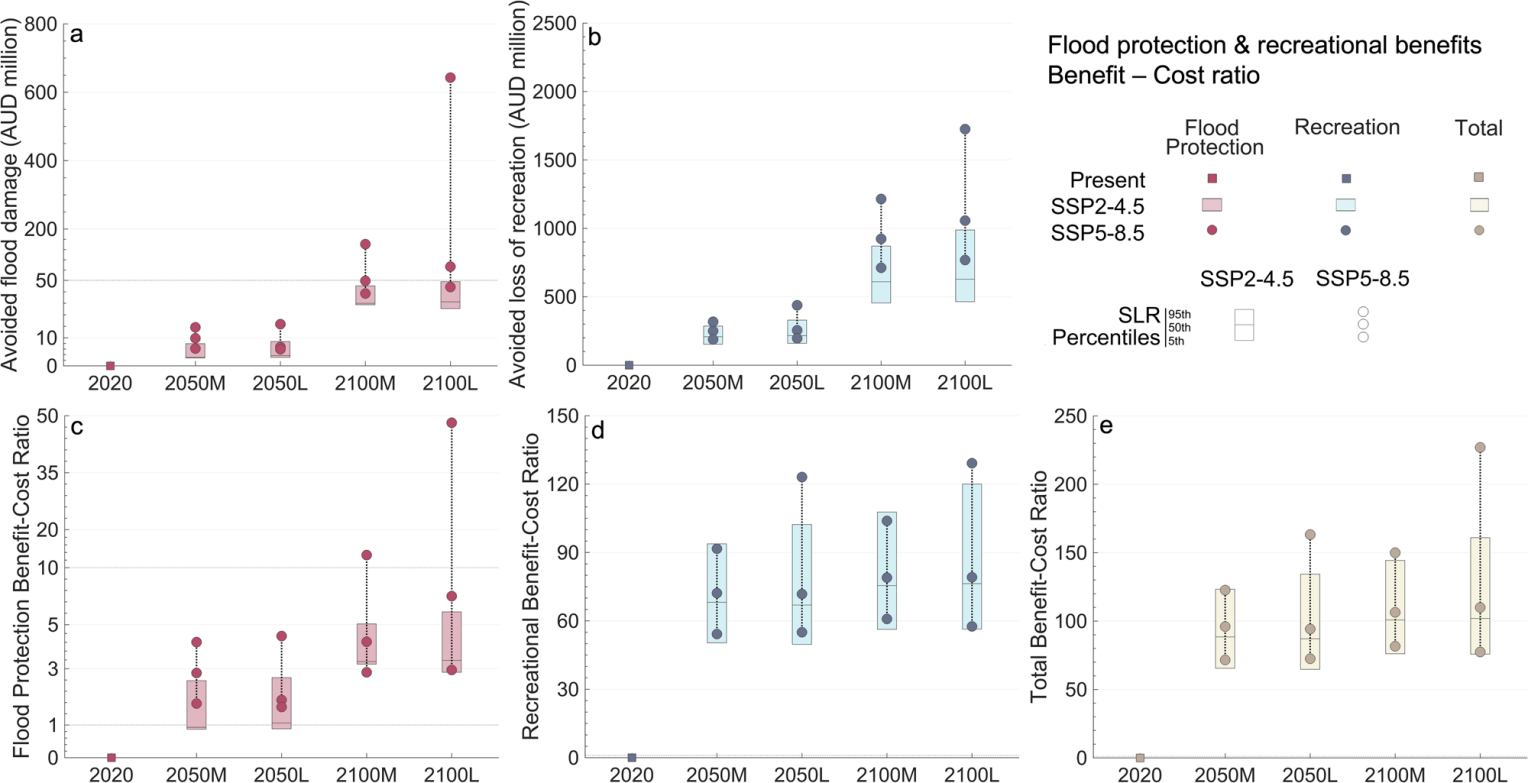
Benefits of maintaining the present-day mean coastline at Narrabeen-Collaroy and benefit-cost ratio. The benefit-cost ratio is a first-pass evaluation of the trade-off between these benefits and the cost of beach nourishment to counteract sea-level rise (SLR) erosion. **a** Flood protection benefits in terms of avoided flood damage (million AUD). **b** Recreational benefits in terms of avoided loss of recreation (million AUD). **c** Cost-benefit ratio considering only flood protection benefits. **d** Cost-benefit ratio considering only recreational benefits. **e** Total cost-benefit ratio. Results are shown for 2020, 2050 and 2100 for two SLR confidence scenarios (medium and low confidence, M and L, respectively), two emissions scenarios (SSP2-4.5 and SSP5-8.5, boxes and circles, respectively) and three SLR trajectories per each confidence scenario and SSP associated with three percentiles of the distribution (5th, 50th, and 95th, horizontal lines of the boxes and circles for the SSP2-4.5 and SSP5-8.5, respectively). Note that to improve visualisation, in **a** and **c** results below 50 million AUD and 10, respectively, are represented in a distant-proportional distorted scale. The plots were created using MATLAB R2022a.

Decision-making on beach maintenance under the pressure of increasingly frequent storms and SLR would benefit from the evaluation of the trade-off between benefits and costs. Figure 7e shows a first-pass benefit-cost ratio comparing the flood protection and recreational benefits of holding the present-day mean shoreline with the associated beach nourishment costs. This implies that in this analysis we only consider erosion due to SLR and limit storm effects to TWL dynamics. As we do not simulate storm erosion, there is no wave dissipation due to profile changes, and flooding occurs above the mean shoreline. The ratio ranges between 65–160 in 2050 and 75–230 in 2100 and is largely due to the recreational benefit (Fig. 7b), which is around an order of magnitude higher than the flood protection benefit (Fig. 7a). The comparison between Fig. 6a and Fig. 7a shows that the greatest flood protection benefits come from maintaining the present-day mean shoreline. This is because the erosion caused by the 30-year storm is small, although this could change for more severe storms. The flood protection ratio (Fig. 7c) suggests that nourishment is cost-effective for all scenarios except for the SSP2-4.5 lowest percentiles in 2050. The avoided flood damage could be up to 4.5 times in 2050 and near 50 times in 2100 the cost of nourishment to mitigate SLR erosion. The recreational ratio can range from 50 to 130 and its high magnitude is because recreation is a perceived good not regulated by the market (Fig. 7d).

## Discussion

As illustrated for Narrabeen-Collaroy, beaches provide significant flood protection benefits that will increase in the future. This process-based analysis allows quantifying the economic value of beaches as natural coastal defences and identifying when erosion due to the combination of SLR and storminess (single storm and cumulative effect due to consecutive back-to-back storms) may result in the greatest flood impact and when management and conservation would enhance the benefits. Knowing how resilient the beaches might be, if they will have a buffer zone to accommodate landward translation due to the compound effect of storms and SLR, and when beach decline may increase flood damage is essential information to plan for successful and cost-efficient adaptation in sandy beaches.

Unlike mangroves and coral reefs, beaches can rapidly adapt to changes in TWLs and continue to provide protection at the storm scale and in the long term. As such, their understanding in the context of adaptation requires approaches that allow modelling the adjustments they experience at different time scales (from hours/days to several decades). At these scales, coastal landscape changes can amplify known impacts (e.g., more flooding) or cause new conditions (e.g., from non-flooding to flooding), which may be related to tipping points^2^. The resilience of beaches allows them to recover from storm erosion during calm periods. For example, at present, a 30-year storm at Narrabeen-Collaroy does not result in an increase in flood damage for the scenario with erosion relative to that without erosion. The situation changes, however, if that storm hits the coast without allowing time for the beach to recover from a previous storm. Storm clusters may cause the beach to exceed its resilience threshold and not fully return to its original condition even if the drivers of change are abated. In the future, SLR erosion will provide a new base coastline for storms to further act on and will contribute to reducing the return period of present storms. If not managed with adaptation, major and irreversible consequences on the beach and the coastal socioeconomic system will occur. Identifying the timing and nature of tipping points can help improve our present understanding of the benefits of beaches for coastal protection, whose expected service life and failure modes need to be quantified to define proper adaptation pathways.

The flood protection benefits of beaches depend on storm characteristics (e.g., intensity and duration); the swash-zone profile shape; the beach condition at the time the storm hits the coastline; the presence of coastal defences; the distribution of terrain heights, land uses and assets; the market value of land and buildings; and the vulnerability of flood exposure to damage. In the future, waves and storm surges are expected to change in some areas^47,48^. This could affect the properties of the storms, which will occur at higher sea levels, thus changing the nearshore wave propagation, the surf and swash zones, and the foreshore slope. Although at an uncertain rate, SLR will likely lead to more frequent flood events and the loss of beach resilience. Chronic dune deterioration can accelerate erosion and increase flooding; and changes in the degree of anthropisation of the coast can have a direct effect on the evolution of the shoreline (e.g., changes in sediment balance, beach rotation or scouring) and on flooding (e.g., changes in overtopping flow or the flooded area). We can also expect a worsening of exposure and vulnerability. The built environment of coastal regions is likely to change with hotspots of in-and-out migration^49^, higher rates of urbanisation^50^ and larger dwellings, even in highly vulnerable regions^51^. Additionally, the market value of property assets can be influenced by supply and demand, or erosion^36,37^. We address some of these aspects in this study, however the analysis of the effect of future changes in storm properties and assets, including their value and vulnerability, would benefit from further research.

Coastal flood and erosion risks will in turn be shaped by adaptation interventions aimed to reduce exposure and vulnerability at some cost. One such intervention is beach nourishment, which has long been the leading coastal protection measure in many countries^52,53^. Compared to grey infrastructure, beach nourishment can have important co-benefits, such as conservation of coastal habitats and provision of aesthetic and recreational values, and provide the flexibility needed for long-term policy strategies to cope with uncertainty. However, feedbacks between the dynamics of human and biogeophysical systems may result in some of these benefits becoming partly offset or reversed in the long run. Research into developed coastlines shows that investments in beach nourishment directly influence coastal housing markets and are capitalised in coastal property values^54^, which in turn may attract a higher density housing development^35,55^. Part of this positive feedback between beach nourishment and coastal development can be extended to other protection policies^56^, which need to be complemented by land-use planning strategies and regulations. Otherwise, the effect of perceived risk reduction together with strong real estate market pressures and the availability of private insurance could result in a maladapted coastline. A comprehensive benefit-cost analysis would require including additional ecosystem services^57^ as beach benefits, long-term effects, ancillary costs^58^, and policies other than beach nourishment, which in the future could become prohibitively expensive in some locations where sand reserves are depleted^36^.

This article presents an approach and results whose signal and order of magnitude provide insights on the real, often unknown value of beaches and on how they could be better managed in the future. A realistic valuation of beach benefits can support land use and management; help understand the trade-offs that may be necessary to achieve short- and long-term goals; and accelerate the application of financial instruments such as parametric insurance or catastrophe and green bonds for beach restoration. This evaluation may also be useful in providing evidence to catalyse private investment that seeks return through investment in beach nourishment-based coastal adaptation and conservation.

## Methods

### Study site

We performed the analysis in the Narrabeen-Collaroy beach system, Sydney, Australia (Fig. 3). The availability of data and calibrated model parameters and the fact that it has been extensively studied makes it an ideal site to showcase the proposed approach. Narrabeen-Collaroy beach is a 3.6 km long embayed beach that is located 20 km north of Sydney and bounded by Long Reef Point to the south and Narrabeen Headland to the north. The beach has a microtidal (spring tidal range of 1.3 m) semi-diurnal tidal regime and it is composed of fine to medium (0.3–0.4 mm) sand. Due to the minimal sediment interactions between the beach, the lagoon, and adjacent beaches, the sediment contained within the embayment can be considered a closed system^59^.

### Replicability

The approach presented here (Fig. 1) is replicable in any other sandy beach provided that coastal climate and topo-bathymetric data, observations, assets value and type, and vulnerability functions are available. Note that climate information can be obtained from global databases and there are vulnerability functions adjusted to building typologies or representative of regions that can be used in case no empirical data are available. Table S1 provides a summary of the data required to implement each step of the methodology. While Narrabeen-Collaroy is an embayed beach, the same modelling chain is applicable to pocket, open and inlet-adjacent beaches. In more complex environments or when required, the 1D hydro-morphodynamic model^60^ can be replaced by its 2DH version to consider local 2DH hydrodynamics that would be otherwise missed (e.g., rips and tidal currents in estuaries) in addition to having a better representation of bathymetry. Long-term processes that may induce coastal landscape changes other than SLR’s such as longshore sediment transport or other sinks or sources can be considered in the profile translation model^61^ by shifting active beach profiles horizontally (e.g., ref. ^25^). We used existing hedonic values (based on market land values and property sales transactions) to estimate the flood protection value and contingent valuation (based on the travel cost method) to measure the recreational value to beach visitors. To illustrate the methodology, we followed a consequences-based approach^62^ and computed flood damage and loss of beach recreation associated with the 30-year storm, so the value of the beach is conditional on that individual event. A more general value could be obtained using a risk aggregated parameter such as the avoided expected annual damage (EAD) or loss of recreational value (e.g., ref. ^31^). The EAD is considered to be the area under the curve defined by the damages associated with storms of different return period. This area could be computed directly after the application of the methodology to more storms, as many more as needed to accurately define the damage curve. For transparency, Table S2 provides for each step of the methodology the underlying assumptions, the uncertainty approaches, and the main limitations. Importantly, the limitations of the data and inherent to the models used in this demonstration do not invalidate the methodology and where better information and tools are available these can be replaced and the methodology will continue to apply.

### Climate data

We obtained hourly offshore wave conditions for the period 1980–2020 from the global wave hindcast GOW2^63^. For the same period of time, we obtained hourly storm surges from the Sydney Port Jackson tide gauge station of the GESLA dataset (https://gloss-sealevel.org) and reconstructed hourly astronomical tides using the harmonic constituents from the TPXO7.2 global model^64^. We used AR6 SLR projections^46^ for the SSP2-4.5 and the SSP5-8.5 emissions scenarios, for medium and low confidence driving processes (that account for uncertainty in ice-sheet mass loss and its regional impact on SLR), for the 5th, 50th and 95th percentiles, and for 2050 and 2100 (Fig. S1). We provide a description of the SLR scenarios and the rationale for their choice in the Supplementary Material. In this analysis we did not use climate change projections of storm surges and wave conditions because of the sign of the change and its uncertainty in the area^47,48^.

### Topo-bathymetric data and non-erodible layer

We used the NSW Marine LiDAR Topo-Bathy 2018 that is available at the Geosciences Australia ELVIS website (https://elevation.fsdf.org.au). This topo-bathymetry is a combined gridded terrestrial (elevation) and subtidal (bathymetry) data at 5 × 5 m (horizontal resolution), Geotifs in GDA 2020 (horizontal datum) to Australian Height Datum (vertical datum) and vertical precision to International Hydrographic Order (IHO) 1B. We used the aerial imagery (World Imagery from Esri) to define a non-erodible barrier to limit the coastline landward displacement according to physical constraints. For deep water, we used the GEBCO Grid, which is an ocean terrain model that provides global coverage of elevation data on a 15 arc-second interval (https://www.gebco.net/data_and_products/gridded_bathymetry_data/).

### Land and building values and land use data

We obtained land values in Narrabeen-Collaroy from the NSW Office of the Valuer General. We used the July 2021 land value, which reflects the real estate market at that date and is based on the analysis of over 67,000 property sales. The land value is the market value of only the land, it does not include the value of buildings or other structures. This information is provided by district in a.csv file at http://valuation.property.nsw.gov.au. Additionally, we consulted real estate websites to obtain the spatial distribution of the built properties prices in the area. We used the NSW Landuse 2017 v1.2 (https://datasets.seed.nsw.gov.au/dataset/nsw-landuse-2017-v1p2-f0ed) to infer the spatially distributed Manning roughness values required for the flood modelling, as in ref. ^65^. In this analysis we did not use projections of land value, building value or land use. We assumed that they would remain constant in the future.

### Nearshore downscaling

We obtained hourly nearshore waves at 200 m-spaced transect ends located throughout the −15 m isobath (Fig. S2) during the period 1980–2020 by efficiently propagating a high-fidelity spectral description of offshore wave climate following ref. ^66^. A representative subset of 500 offshore sea states characterised by their full frequency-direction spectrum was obtained from the GOW2 dataset using the maximum dissimilarity selection algorithm^67^. Then, we propagated offshore sea states using the nearshore spectral wave model SWAN^68^ to the nearshore points. We designed two SWAN grids for accurately capturing the wave propagation processes: a general grid of 500 m resolution extending 100 km alongshore and forced by the GOW2 points; and a detailed grid of 50 m resolution covering the study site and nested to the general grid (Fig. S3). Selected offshore sea states and their nearshore concomitants formed an interpolation basis from which we reconstructed the complete nearshore wave climate using radial basis functions^69^.

### Long-term topo-bathymetry update

We considered SLR to be the main driver of long-term morphological changes, as we did not identify any significant long-term trend from the subaerial beach volume time series analysis (Fig. S4). First, we transferred the present-day topo-bathymetry (which we assumed to be in equilibrium, Fig. S5) and the non-erodible barrier to 10 m-spaced cross-shore transects extending from the 5 m contour line to the −15 m isobath. Then, for every SLR scenario considered, we applied the profile translation model ShoreTrans^61^. ShoreTrans is a rules-based sediment budgeting model that updates surveyed profiles based on kinematic translations associated with the modelled processes. In the case of SLR, profiles are shifted upwards and landwards (Fig. S6) in order to assure a net zero volume change while eventually considering local scour due to non-erodible defences through heuristic rules^70^. We generated a cloud of 10 m alongshore × 1 m cross-shore points for every SLR scenario that we interpolated in ArcGIS to update the present-day topo-bathymetry. Figures S7 and S8 show the long-term topo-bathymetric changes for the scenarios considered.

### Storm definition and surf-zone storm modelling

From the 32-year database of nearshore waves and water levels, we defined the storm once in 30 years as a reference. We first calculated an empirical TWL as the sum of storm surges, astronomical tides, wave setup and infragravity swash following ref. ^71^. We fitted the annual maxima to an empirical distribution and obtained the reference storm as the observed event closer to the 30-year return period TWL. After identifying the peak of the storm through the TWL, the duration was defined by the period of time in which the significant wave height exceeded a threshold^72^. We set the threshold based on the monthly running mean of the significant wave height (Fig. S9). We used hourly nearshore sea states and water levels during the storm duration to force the hydro-morphodynamic model XBeach^60^ in a 1D surfbeat configuration with the calibrated parameters obtained from ref. ^73^. Although the beach could have certain alongshore variability in terms of calibration^74^, for simplicity we have considered a representative set of parameters. We performed several simulations for every SLR scenario considering present-day or long-term updated profiles and by switching the morphodynamics in the XBeach calculation on and off (Fig. S10), considering the non-erodible barrier that limits erosion. From the simulations, we obtained the time series of water excursion and bed evolution at the 200 m-spaced transects during the reference storm.

### Short-term topo-bathymetry update

We incorporated storm erosion into present-day and long-term updated topo-bathymetries by transferring the process-based profile kinematics calculated in the 200 m-spaced profiles. Based on the spatial uniformity of the beach, we transferred the cross-shore bed changes at the 200 m-spaced profiles to the 10 m-spaced profiles using distance-based interpolation throughout the active zone. Then, from the 10 × 1 m point cloud, we generated a surface and superimposed the changes to the base present-day or long-term topo-bathymetry. To analyse the effects of the initial morphological condition of the beach, we calculated the cross-shore bed changes and the associated topo-bathymetry at the peak of the TWL and at the end of the storm (storm and poststorm beach conditions, respectively). The poststorm beach condition represents a situation of storm clustering in which the beach fails to recover between back-to-back storms. Figures S11 and S12 show short-term topo-bathymetric changes under the scenarios considered for storm and poststorm conditions, respectively.

### Coastal flood modelling

For every scenario, we obtained maximum flood extents and depths in a 5 m resolution grid (Fig. S13a) using the process-based 2D flood model RFSM-EDA^75^. RFSM-EDA is an efficient inland flood-spread model that solves the simplified shallow water equations in a subgrid representation of the topography. We forced the model with the temporal evolution of the waterfront elevation (TWL) calculated with XBeach while changing the underlying emerged topography based on the scenario of SLR and morphological update.

### Flood protection value assessment

We computed the flooded area and the flood damage using ArcGIS. To calculate flood exposure, we digitised each land parcel in the Narrabeen and Collaroy districts and assigned them a unique value per m^2^, computed using the street name, house, area, and land value fields of the NSW Office Value General for each land parcel (Fig. S14a). We also digitised each building and assigned them a unique value per m^2^, computed considering the floor area, the number of floors (estimated with Google Street View) and the spatial distribution of real estate prices per m^2^ (Fig. S14b). Likewise, we digitised the roads in the area. We removed the buildings from the land parcel dataset to avoid double counting. We combined the three spatially distributed datasets with the flood maps and multiplied the flooded area of each land parcel and building by their values per m^2^. To transform flood exposure into flood damage, we applied different vulnerability functions. For land parcels flooded above a threshold of 0.2 m, we applied a relative damage factor of 1% representing clean up labours (consistent with average disaster costs estimates in Australia^76^). For buildings, we applied average relative depth-damage functions for residential and commercial properties in Oceania^77^. For roads, we adopted the recommendations of the RAM model^78^. For the road area flooded above the threshold of 0.3 m, we applied an absolute damage factor of 3.71 AUD/m^2^, which we updated considering inflation. Additionally, we considered indirect damages to roads (e.g., traffic delays) to be in the order of 30% of the direct damages. We applied this procedure to the scenarios without erosion (static approach) and with erosion (dynamic approach). We computed the beach protection value in terms of avoided flood damage by subtracting the flood damage of the scenarios without erosion from the flood damage of the scenarios with erosion. We assumed that in the future asset values will not change and the vulnerability functions used will remain valid.

### Beach recreational value assessment

We computed the recreational value using the aggregate annual value of beach recreation provided by the Sydney Beaches Valuation Project^79^. In that project, the travel cost method was applied to obtain the total consumer surplus of visits in Narrabeen-Collaroy (118.1 million AUD per year). This cash flow represents the extra uncosted utility or benefit that people receive from physically using the resource every year. We capitalised the annual value of beach recreation (economic value) into its present value (accounting value) considering inflation and a discount rate of 4% following ref. ^31^. We obtained the value of beach recreation per m^2^ by dividing the accounting value by the present-day beach area. We multiplied the value of beach recreation per m^2^ by the available beach area for the scenarios with erosion (dynamic approach) and without erosion (static approach). We computed the beach recreational value in terms of avoided loss of beach recreation if the present shoreline was maintained by subtracting the recreational value of the scenarios with erosion from the recreational value of the scenarios without erosion. We assumed that each m^2^ provides the same recreation and is worth the same; the discount rate is fixed and does not vary based on the risk of loss of available beach area; and users will not change their preferences in the future.

### Benefit-cost analysis

We obtained a simple benefit-cost ratio by comparing the benefits provided by Narrabeen-Collaroy associated with SLR with the cost of maintaining the present-day mean shoreline through nourishment. We computed the benefit as the avoided flood damage and loss of beach recreation which would result from not allowing the mean shoreline to be eroded by SLR. We computed the cost of maintaining the present-day mean shoreline by combining the eroded volumes from the scenarios with SLR erosion with the total unit cost for beach nourishment (30 AUD/m^3^) estimated by the ref. ^80^, which we updated considering inflation. In the ratio, we considered only the part of the beach eroded by SLR because adaptation to SLR is usually planned and risk reduction or adaptation to a storm is usually a reactive response. We assumed sediment availability, one nourishment intervention per scenario, and a single price for regeneration campaigns that is constant over time, which may not be the case.

### Variance partitioning analysis

We performed a four-factor, ANOVA-based variance decomposition where SSPs, SLR percentiles and modelling approaches (dynamic approach with erosion and static approach without erosion) were the uncertainty sources. We used the n-way analysis of variance (anovan) MATLAB function. The relative contribution of a component to the total TWL, flooded area and flood damages uncertainty is expressed by the fraction of each component’s variance to the total variance. We set the nesting relationships among the SSPs and SLR percentiles so that their interactions capture the interaction between the modelling approach and the SLR dimensions but not between the SLR dimensions themselves.

## Supplementary information

Supplementary Information

## Data Availability

The data generated in this study has been deposited in the Zenodo database under accession code 10.5281/zenodo.7939983.

## Code availability

The SWAN model can be downloaded from https://swanmodel.sourceforge.io/download/download.htm. The XBeach source code is hosted at https://oss.deltares.nl/web/xbeach/release-and-source. The ShoreTrans model is accessible from https://github.com/jakmccarroll/ShoreTrans.
