# Supplementary material for: Demonstrating the value of beaches for adaptation to future coastal flood risk
Source: Nat Commun. 2023 Jun 12;14:3474. doi: 10.1038/s41467-023-39168-z (PMC10261140; doi:10.1038/s41467-023-39168-z)
Supplement: Supplementary file 1 — Supplementary Information [file 41467_2023_39168_MOESM1_ESM.pdf]

Supplementary Material for:

Demonstrating the value of beaches for adaptation to future coastal flood risk

Alexandra Toimil[1,2], Iñigo J. Losada[1], Moisés Álvarez-Cuesta[1], Gonéri Le Cozannet[2]

[1]IHCantabria - Instituto de Hidráulica Ambiental de la Universidad de Cantabria, Isabel Torres 15, 39011, Santander, Spain.

[2]Bureau de Recherches Géologiques et Minières "BRGM", French Geological Survey, 3 Avenue Claude Guillemin, CEDEX, 45060 Orléans, France

Corresponding author: Alexandra Toimil (toimila@unican.es)

Key features of the proposed methodology

Steps	Approach	Data
Nearshore downscaling	Hybrid downscaling combining wave generation (SWAN, [1]) and statistical ([2; 3]) modelling.	Offshore wave, storm surge, astronomical tide, and mean sea-level rise (SLR) data. Offshore bathymetry.
Long-term topo-bathymetry update	Application of a profile translation model (ShoreTrans, [4]).	SLR data. Present topo-bathymetry. Aerial imagery.
Storm definition and surf-zone storm modelling	Obtention of the present-day 30 years storm empirically. Associated surf-zone 1D hydro- and morphodynamic modelling (XBeach, [5]).	Offshore wave, storm surge, astronomical tide, and (SLR) data. Aerial imagery. Present topo-bathymetry. Long-term topo-bathymetries.
Short-term topo-bathymetry update	Application of a distance-based interpolation method to translate the storm morphological changes calculated at the transects to the topo-bathymetry.	Present-day 30-year storm morphodynamics at the peak of the storm and after the passing of the storm (outputs from XBeach). Present topo-bathymetry. Long-term topo-bathymetries.
Coastal flood modelling	Hydraulic modelling (RFSM-EDA, [6]).	Present-day 30-year storm hydrodynamics (output from XBeach) and SLR data. Land use data. Present topo-bathymetry. Short-term topo-bathymetries.
Flood protection value assessment	Obtention of the avoided flood damage by subtracting the flood damage of the scenarios without erosion from the flood damage of the scenarios with erosion We computed flood damages by combining flooded hedonic values (based on land values and property sales transactions) and vulnerability functions ([7; 8]).	Flood maps. Land parcels value, area and type Building value, area and type. Infrastructures (e.g., roads). Vulnerability functions.
Beach recreational value assessment	Obtention of the avoided loss of recreation by subtracting the recreational value of the scenarios with erosion from the recreational value of the scenarios without erosion. We measured beach recreation using contingent valuation (travel cost method) following [9].	Available beach area. Value of beach recreation per unit area.
Benefit-cost proxy	Comparison of the avoided flood damage and loss of recreation associated with SLR with the cost of holding the mean shoreline (excluding seasonal and inter-annual variations) with nourishment.	Avoided flood damage. Avoided loss of beach recreation. Cost of nourishment.

Table S1 Summary of modelling approaches and data required for their application associated with the different steps of the methodology.

Steps	Assumptions	Uncertainty	Limitations
Nearshore downscaling	SLR is the most uncertain climate driver of coastal flooding and erosion. Wave conditions and storm surges will not change in the future.	Range of SLR scenarios (from 0.100 m to 2.321 m) that results from considering 2 emissions scenarios, SLR driving processes associated with 2 confidence levels, and	Inherent to the skill of the wave generation model to simulate physical processes (e.g., wave diffraction poorly represented).

	The bathymetry does not change beyond the depth of closure.	3 trajectories associated with 3 percentiles ([10]). Discarding the use of wave and storm surge projections because of the sign of the change and its uncertainty in the area ([11; 12]). Propagation of the full frequency-direction spectra rather than the aggregated parameters to have a high-fidelity description of wave climate ([13]).	
Long-term topo-bathymetry update	SLR is the main driver of long-term morphological changes, as we did not identify any significant long-term trend from the subaerial beach volume time-series analysis. The present-day topo-bathymetry is in equilibrium.	Associated with SLR, as we developed one long-term topo-bathymetry for each SLR scenario.	Inherent to the profile translation model, as the profile kinematics considered are a simplification of reality.
Storm definition and surf-zone storm modelling	The peak of the storm depends on wave and sea-level conditions and the duration of the storm depends on wave conditions exceeding a threshold. A set of calibration parameters ([14]) is representative for all the profiles.	30-year storm determined empirically, as we had ≈30 years of wave data and so we avoided extrapolation. Associated with SLR, as we combined the present-day 30 years storm with each SLR scenario.	Inherent to the storm definition method and the skill of the hydro-morphodynamic model to simulate physical processes (e.g., beach flattening). As we run XBeach in its 1D version, we missed 2DH hydrodynamics that can occur locally (e.g., rips).
Short-term topo-bathymetry update	The present-day topo-bathymetry is in equilibrium.	Associated with SLR and the two magnitudes of storm erosion (at the peak of the storm and after passing the storm), as we developed two short-term topo-bathymetries over each long-term topo-bathymetry.	Inherent to the skill of the storm morphodynamic model and the topo-bathymetry interpolation procedure.
Coastal flood modelling	The short-term topo-bathymetry that incorporates erosion at the peak of the storm represents the baseline for the storm condition (flooding over an uneroded beach). The short-term topo-bathymetry that incorporates erosion at the passing of the storm represents the baseline for the poststorm condition (flooding over a beach previously eroded by a recent storm). Land uses will not change in the future.	Associated with SLR and the two beach conditions (storm and poststorm), as we obtained two flood maps for each SLR scenario combined with the present-day 30-storm considering two short-term topo-bathymetries per each SLR scenario.	The hydraulic model is forced with hydrodynamic conditions computed externally and runs over an emerged topo-bathymetry updated externally (decoupled processes). Inherent to the skill of the hydraulic model to simulate physical processes (e.g., it solves a simplification of the shallow water equations in large cells).
Flood protection	The value of land and buildings will not change in the future.	Associated with SLR and the two beach conditions, as we obtained the avoided flood	Inherent to the vulnerability functions.

value assessment	The<br>The vulnerability functions will not change in the future.<br>Land uses will not change in the future.<br>Future damage (damage subject to repair or replacement) occur due to the present-day 30-year storm over SLR, as we found that SLR inundation does not reach any buildings or infrastructures.<br>There is no loss of economic activity (loss of profit) associated with the flooding of commercial buildings.	damages for each scenario (storm and poststorm conditions combined with SLR scenarios).	
Beach recreational value assessment	Each unit area provides the same recreation and is worth the same.<br>Users will have the same preferences in the future.<br>The discount rate is fixed and does not vary based on the risk of loss of beach area.	Associated with SLR and the two beach conditions (storm and poststorm), as we obtained the avoided loss of beach recreation for each scenario (storm and poststorm conditions combined with SLR scenarios).	Inherent to the approach used to compute the accounting value of beach recreation.
Benefit-cost proxy	Sediment availability.<br>One nourishment intervention per scenario.<br>A single price for regeneration campaigns.<br>The cost of nourishment will not change in the future.	Associated with SLR, as we obtained the benefit-cost proxy for each SLR scenario. Storms are part of the TWL but no storm erosion is considered.	First-pass benefit-cost ratio that simply results from the quotient of the value of the beach and the value of its conservation.

Table S2 Summary of assumptions, uncertainty treatment approaches and main limitations associated with the different steps of the methodology.

Mean sea-level rise scenarios considered

One of the main sources of uncertainty in future climate is mean sea-level rise (SLR). We have chosen a set of SLR scenarios that cover a wide range of values and combined them with the same storm. This wide range is given by considering: 2 emissions scenarios formulated in terms of shared socioeconomic pathways and their associated level of radiative forcing (SSPx-y) to understand the sensitivity of our decisions to different futures; 2 confidence levels to consider SLR-driving processes of medium and low confidence; and, for each combination of emissions-confidence scenarios, 3 potential SLR trajectories associated with 3 percentiles of the distribution of the results of the climate models included in the AR6 ([10]). For a given scenario, each SLR trajectory therefore shows an evolution of the SLR over time with a different acceleration rate.

As for time horizons, we consider 2050 and 2100. They are two milestones given by the IPCC and adopted by users for adaptation needs. There is ongoing research to assess the suitability of these milestones from surveys conducted to stakeholders. [15] showed that 2100 is the time horizon most widely used for SLR planning in many countries, and that it is followed by 2050.

For both 2050 and 2100, we consider 12 different SLR values that cover a range of futures that expands from 0.100 m (SSP2-4.5 medium-confidence 5th percentile) to 2.321 m (SSP2-4.5 low-confidence 95th percentile). We show all these values in **Table S3** (medium confidence AR6 SLR regional projections near the study beach) and in **Table S4** (the same as in **Table S3** but for low confidence).

The emissions scenarios considered are the AR6 SSP2-4.5 and SSP5-8.5, which correspond to temperature increases in 2100 of 2.7°C and 4.4°C, respectively. The choice of these scenarios is due to the fact that it has been recognised in the literature that their predecessors in the AR5 (RCP4.5 and RCP8.5) are the most widely used in many countries for SLR adaptation planning ([15]).

- The SSP2-4.5 is an intermediate scenario. CO_2 emissions are near current levels before starting to decline by 2050, but do not reach zero by 2100. Socio-economic factors follow their historical trends, with no significant change. Progress toward sustainability is slow, with disparate development and income growth. In 2100, the global mean temperature is 2.7°C warmer than pre-industrial levels.
- The SSP5-8.5 is a very high emissions scenario. Current levels of CO_2 emissions roughly double by 2050. The economy grows rapidly at the cost of the exploitation of fossil fuels and energy-intensive lifestyles. In 2100, the global mean temperature is 4.4ºC warmer than pre-industrial levels.

The range of SLR values resulting from the combination of emissions scenarios and percentiles covers the mean values of the SSP1-2.6, SSP2-4.5, SSP3-7.0, and SSP5.8.5. For instance, the 5th percentile of the SSP2-4.5 is slightly higher than the 5th percentile of the SSP1-2.6 (0.100 m vs 0.081 in 2050; and 0.305 m vs 0.179 m in 2100 considering medium confidence processes) and lower than the 50th percentile of the SSP1-2.6 (0.176 m in 2050; and 0.391 m in 2100 considering medium confidence processes). The same applies for low confidence processes and for the SSP3-7.0 vs the SSP5-8.5.

	**SSP2-4.5**			**SSP5-8.5**		
	Medium confidence					
	5th	50th	95th	5th	50th	95th
**2050**	0.100	0.198	0.338	0.135	0.234	0.378
**2100**	0.305	0.532	0.941	0.496	0.780	1.301

Table S3 Medium-confidence AR6 projections of mean sea-level rise near the Narrabeen-Collaroy beach system.

	**SSP2-4.5**			**SSP5-8.5**		
	Low confidence					
	5th	50th	95th	5th	50th	95th
**2050**	0.100	0.205	0.423	0.135	0.243	0.556
**2100**	0.307	0.545	1.172	0.496	0.899	2.321

Table S4 Low-confidence AR6 projections of mean sea-level rise near the Narrabeen-Collaroy beach system.

The IPCC has acknowledged in its last 3 assessments reports that the main uncertainty in SLR projections is due to the limited confidence in the modelling of future melt dynamics of the Antarctic and Greenland Ice Sheet. However, it was not until the AR6 that the IPCC has presented for the first time a low-likelihood-high impact scenario (LLHI scenario; also referred to in the literature as "high-end" scenario, [16], and "H++" scenario, [17]) showing a SLR of 1.7 m by 2100 and more than 15 m by 2300 that could not be excluded under high emissions ([10]). Additionally, research suggests that LLHI scenarios can be useful for decision makers considering time scales up to centennial and having high risk aversion or low uncertainty tolerance ([18]). In these cases, the emerging practice is to consider likely scenarios together with low-likelihood-high impacts (LLHI) scenarios ([15]; [19]). Further, there is evidence that LLHI scenarios are used for virtual stress tests on critical infrastructure (e.g., Thames river barrier, or the Netherlands; [17]). In summary, the reasons for using these LLHI scenarios in this study are that: (1) they cannot be excluded yet; and (2) they can be used for some coastal adaptation problems.

Table S5 describes the differences in the medium and low confidence AR6 SLR projections in terms of Greenland and Antarctic ice-sheet mass loss processes. We adapted this table from the Table 9.7 of AR6 Chapter 9 on Ocean, Cryosphere and Sea Level Change ([10]).

**Driver of GMSL or RSL change**	**AR6 projection method**
Greenland Ice Sheet (excluding peripheral glaciers)	Medium-confidence processes up to 2100: Emulated Ice Sheet Model Intercomparison Project for CMIP6 (ISMIP6) simulations ([20])<br>Low-confidence processes: Structured expert judgement ([21])
Antarctic Ice Sheet (excluding peripheral glaciers)	Medium-confidence processes up to 2100: p-box including (i) Emulated ISMIP6 simulations ([20]); and (ii) Linear Antarctic Response Model Intercomparison Project (LARMIP-2) simulations ([22]) augmented by AR5 surface mass balance model.<br>Low-confidence processes: (i) Single-ice-sheet-model ensemble simulations incorporating marine ice cliff instability ([23]); and (ii) structured expert judgement ([21])

Table S5 Methods used to project the contribution of Greenland and Antarctic ice-sheet mass loss to global mean sea levels (GMSL) and relative sea level (RSL) change in the SSPs and warming-level-based projections of GMSL, RSL and extreme sea level change. Adapted from Table 9.7 of AR6 Chapter 9 on Ocean, Cryosphere and Sea Level Change ([10]).

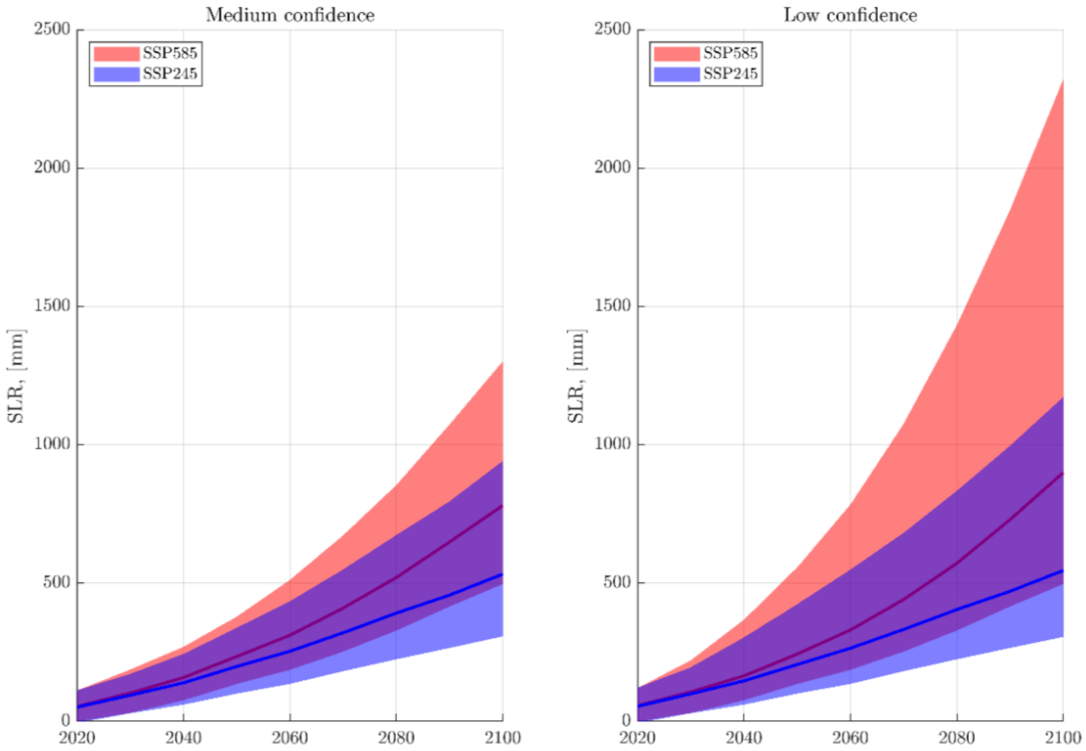

Figure S1. SLR trajectories for the medium and low confidence scenarios of the IPCC AR6 projections. SLR projections corresponding to the 50% SLR percentile (solid lines) and 90% confidence band (defined by the 5th and 95th percentiles) for the SSP245 and SSP585 of the medium and low confidence scenarios of the IPCC AR6 projections.

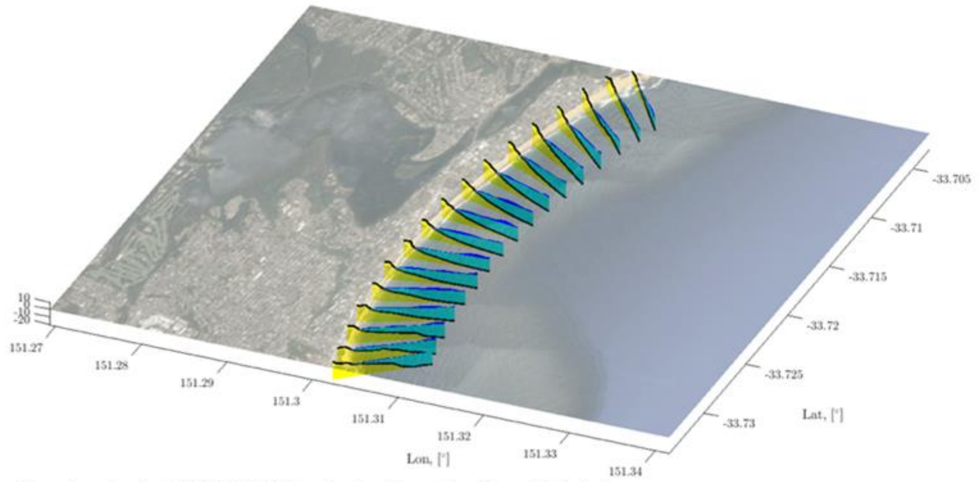

Maps data: Google, © 2022 CNES/Airbus, Landsat /Copernicus, Maxar Technologies

Figure S2. **Computational transects along Narrabeen-Collaroy**. Shoreline discretization of 200 m-spaced transects until the -15 m isobath used for the XBeach TWL modelling. Figure created in MATLAB R2022a.

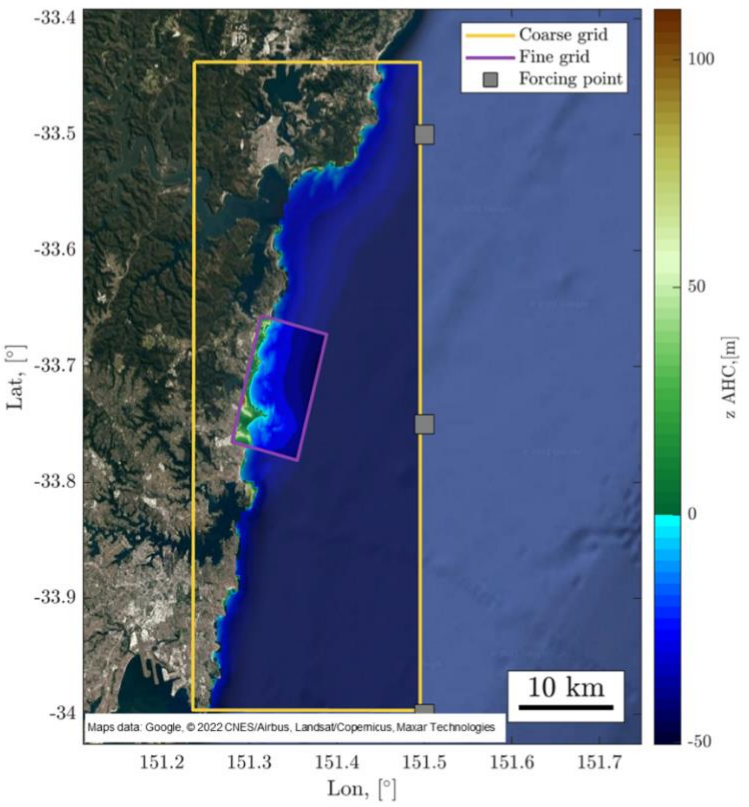

Figure S3. Grid boundaries for the nearshore wave propagation. Computational domain for the SWAN propagation of the nearshore downscaling, highlighting the location of the GOW2 forcing points, the 500x500m coarse resolution grid and the nested 50x50m fine resolution grid. Figure created in MATLAB R2022a.

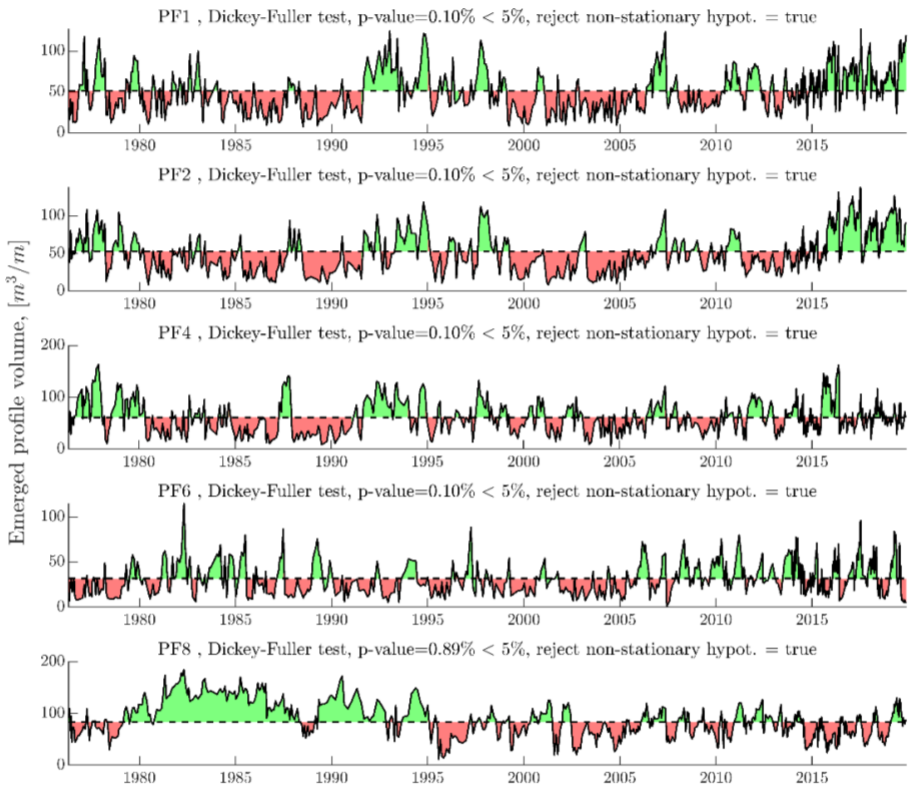

Figure S4. Time-series of subaerial beach volume in different profiles. The dashed line represents the mean volume of the time-series that splits accretion periods (in green) and erosion periods (red). The results the Dickey-Fuller test on the data are also shown in each profile. The existence of unit root hypothesis is rejected in every case, and thus the time series can be considered stationary. For this analysis we have used the data of [24]. Figure created in MATLAB R2022a.

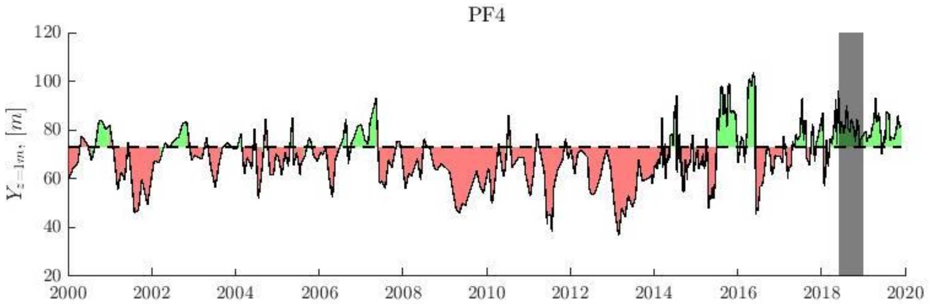

Figure S5. Time-series of shoreline position (z=1m AHD) in a central profile (PF4). The grey shaded area corresponds to the acquisition period of the reference 2018 LiDAR topo-bathymetry. During the acquisition period July-December 2018, the shoreline position is close to the mean value (dashed line) meaning that no significant erosion or accretion event is captured in the reference topo-bathymetry, which is assumed to be in equilibrium. Figure created in MATLAB R2022a.

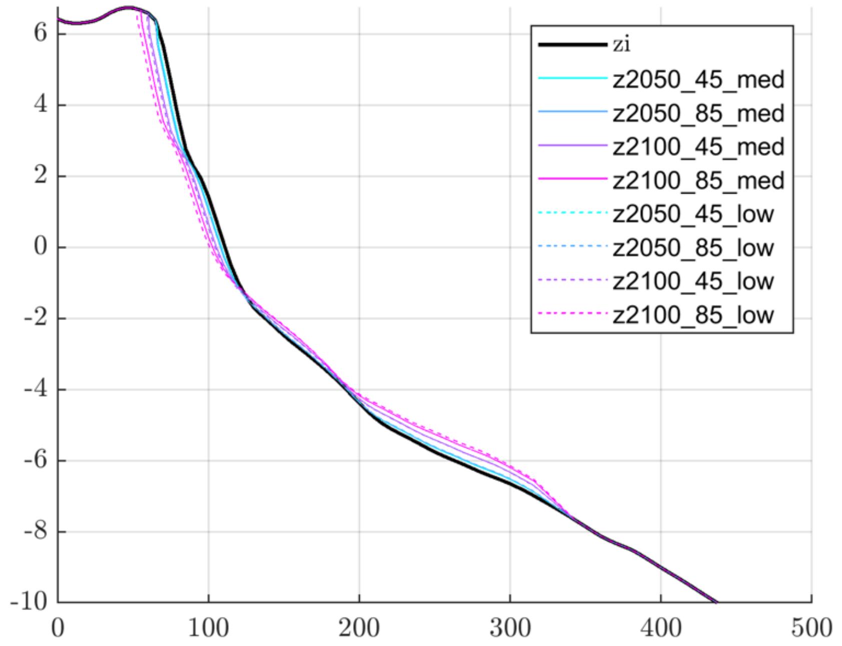

Figure S6. Long-term morphodynamic evolution in one of the transects for different time-horizons and SLR scenarios. The black line corresponds to the present-day geometry. Solid lines represent medium confidence SLR scenarios and dashed lines represent low confidence SLR scenarios. Figure created in MATLAB R2022a.

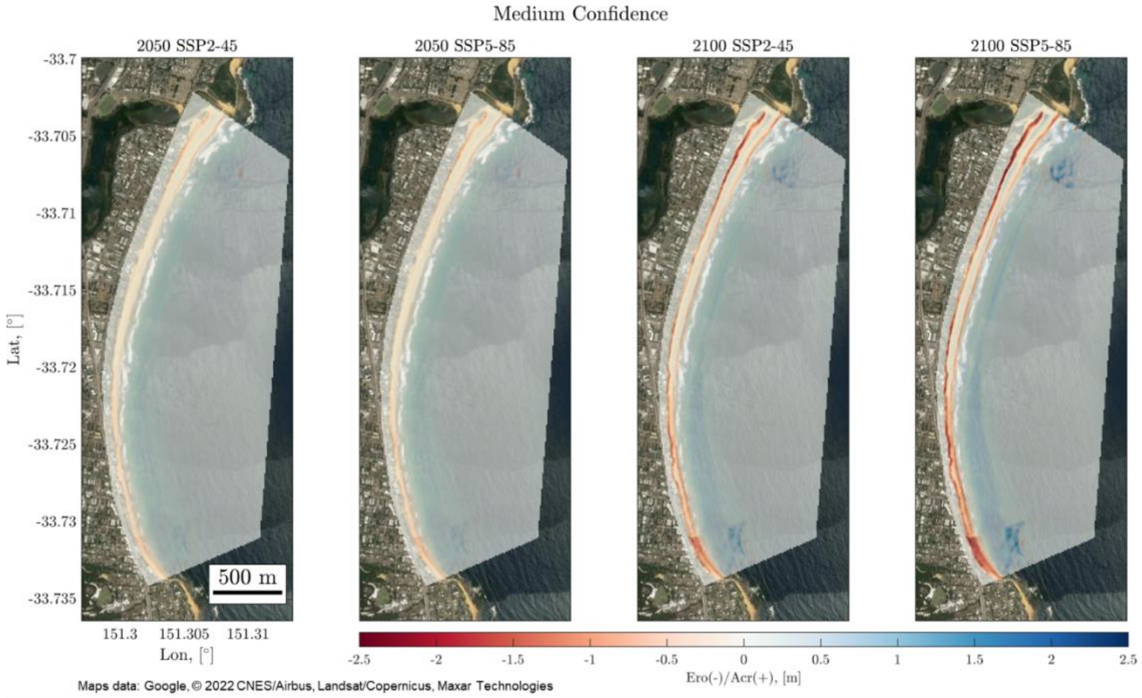

Figure S7. Long-term topo-bathymetric changes for the medium confidence SLR scenarios. From left to right, 2050 SSP2-4.5, 2050 SSP5-8.5, 2100 SSP2-4.5 and SSP5-8.5. Changes are calculated with respect to the present-day topo-bathymetry. Figure created in MATLAB R2022a.

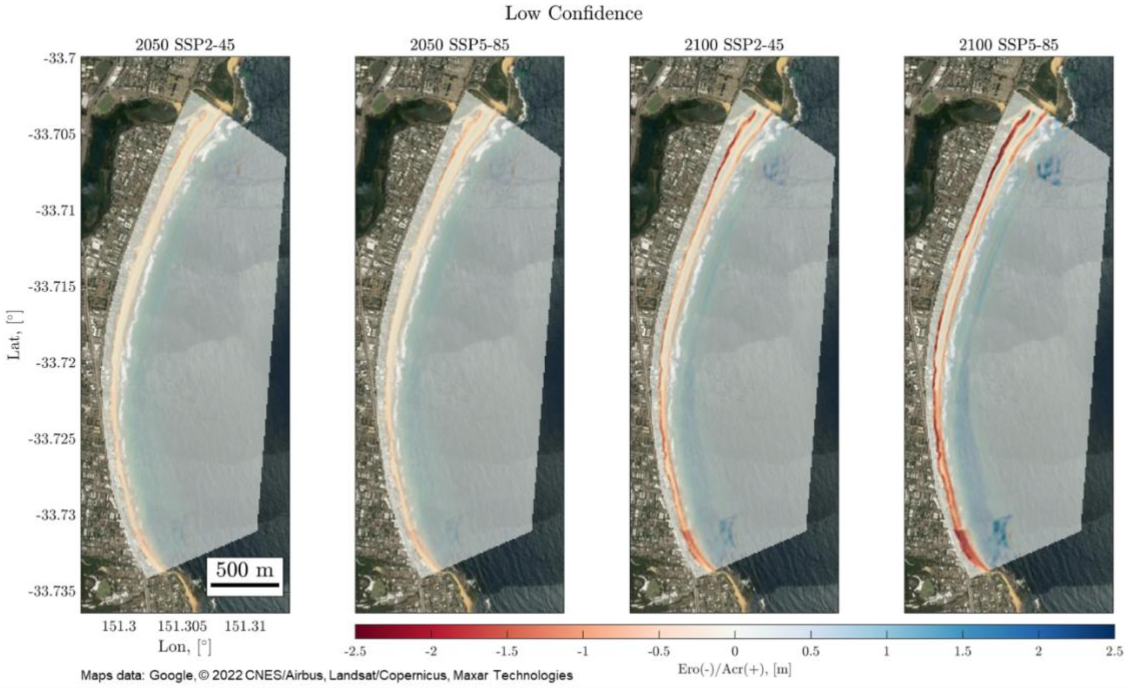

Figure S8. Long-term topo-bathymetric changes for the low confidence SLR scenarios. From left to right, 2050 SSP2-4.5, 2050 SSP5-8.5, 2100 SSP2-4.5 and SSP5-8.5. Changes are calculated with respect to the present-day topo-bathymetry. Figure created in MATLAB R2022a.

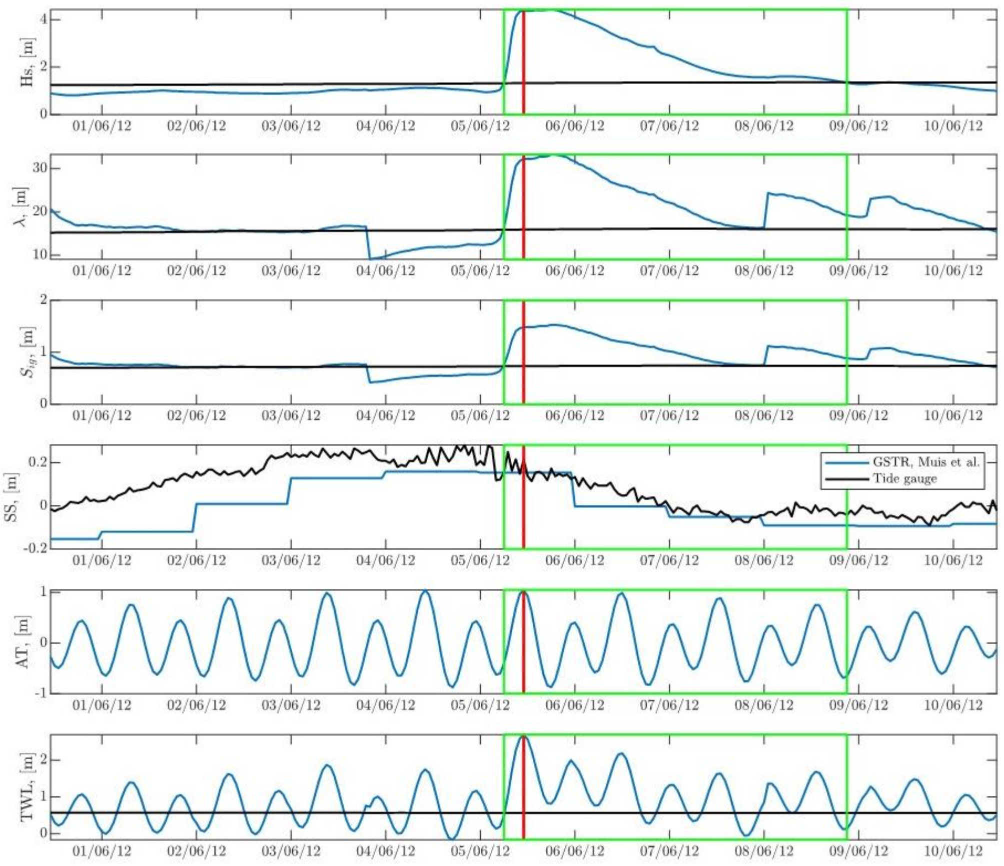

Figure S9. Storm duration definition based on downscaled dynamics. From top to bottom, time series of significant wave height (Hs, [m]), wave length (λ, [m]), infragravity swash (S_{ig}, [m]), storm surge (SS, [m]), astronomical tide (AT, [m]) and total water levels (TWL, [m]). The red line represents the TWL peak and the green windows highlights the storm duration. Figure created in MATLAB R2022a.

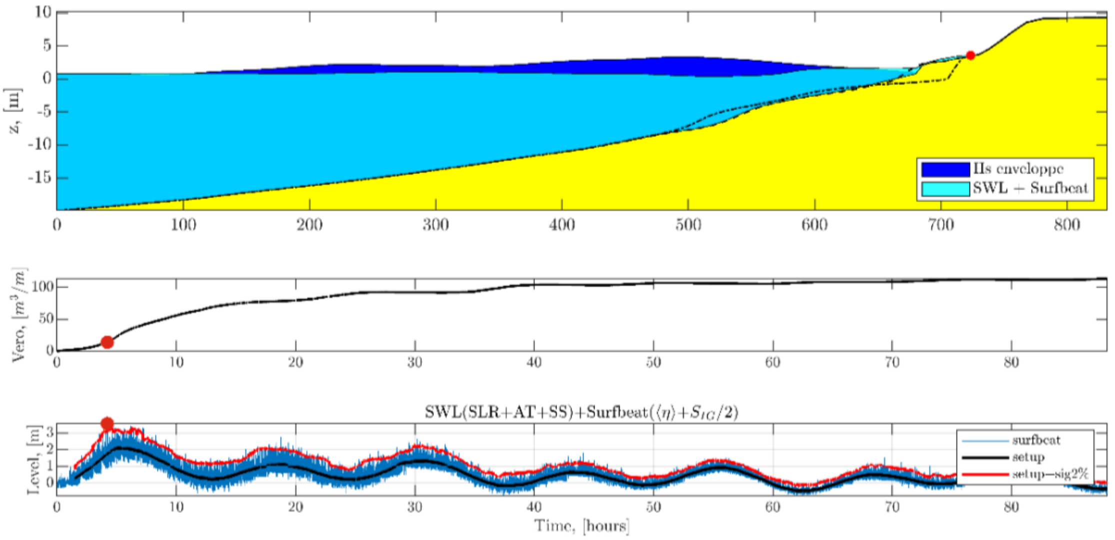

Figure S10. 1D modelling of storm impacts with XBeach at one of the transects for the present-day scenario (without SLR). In the top panel, the wave group envelope is represented in blue, in cyan the still water level (SWL) plus the infragravity swash contribution and in yellow the profile geometry at the point when maximum surfbeat is reached during the simulation. The dashed line is the initial profile geometry while the dashed-dotted line is the final geometry at the end of the simulation. The intermediate panel represents the time history of subaerial eroded volume and the red dot corresponds to the subaerial eroded volume at the moment when the maximum surfbeat occurs. The lower panel correspond to the time series of set-up (black line), surfbeat (blue line) and set-up plus and the infragravity swash of 2% exceedance probability (red line). The red dot highlights the instant when the maximum surfbeat occurs. Figure created in MATLAB R2022a.

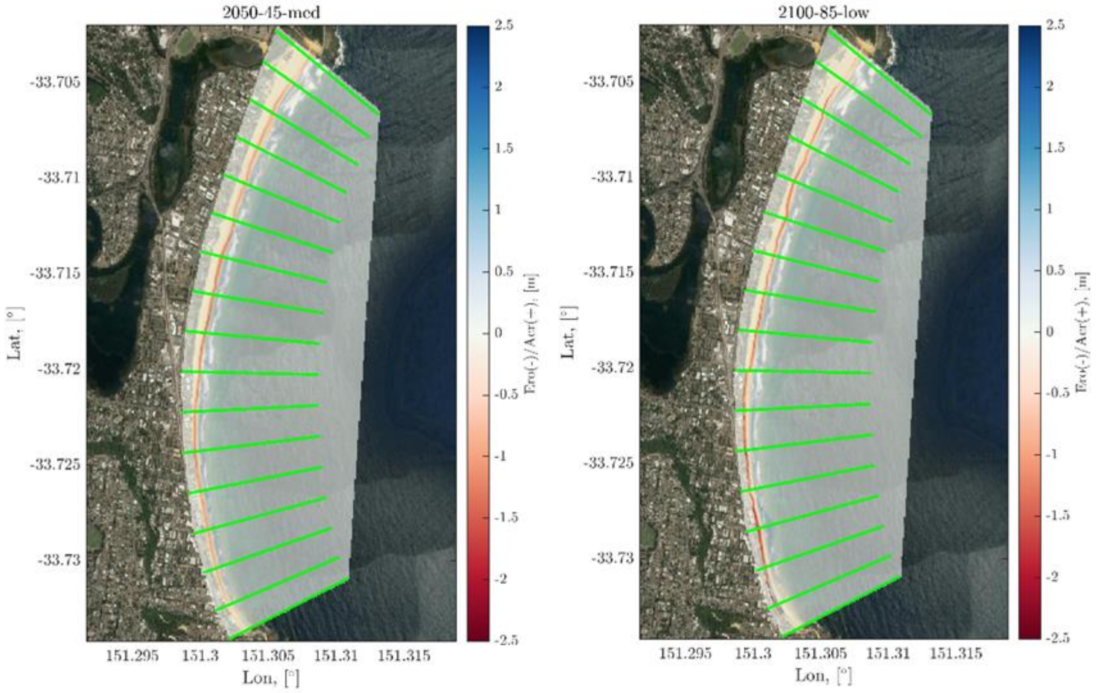

Maps data: Google, © 2022 CNES/Airbus, Landsat/Copernicus, Maxar Technologies

Figure S11. Storm-induced topo-bathymetric changes. The extreme event is acting on the long-term modified topo-bathymetry at two different SLR scenarios, 2050 SSP2-4.5 medium confidence in the left panel and 2100 SSP5-8.5 low confidence in the right panel**.** The changes are calculated with respect to the updated long-term topo-bathymetry. Figure created in MATLAB R2022a.

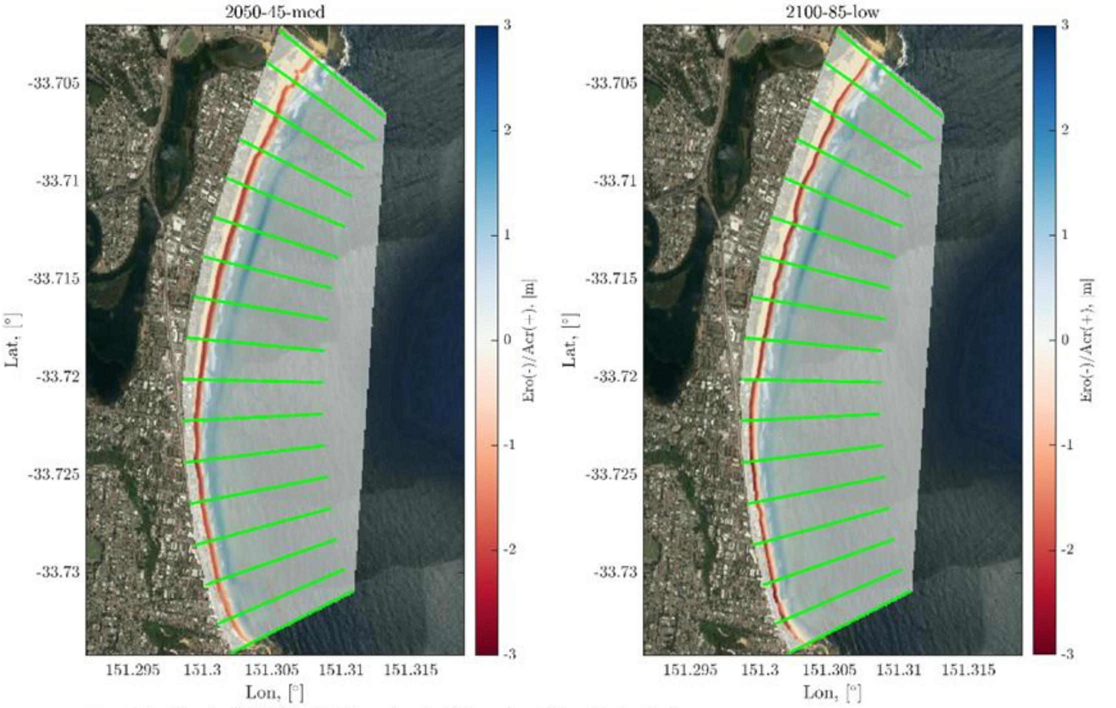

Maps data: Google, © 2022 CNES/Airbus, Landsat/Copernicus, Maxar Technologies

Figure S12. Post-storm-induced topo-bathymetric changes. The extreme event is acting on the long-term modified topo-bathymetry at two different SLR scenarios, 2050 SSP2-4.5 medium confidence in the left panel and 2100 SSP5-8.5 low confidence in the right panel. The changes are calculated with respect to the updated long-term topo-bathymetry. Figure created in MATLAB R2022a.

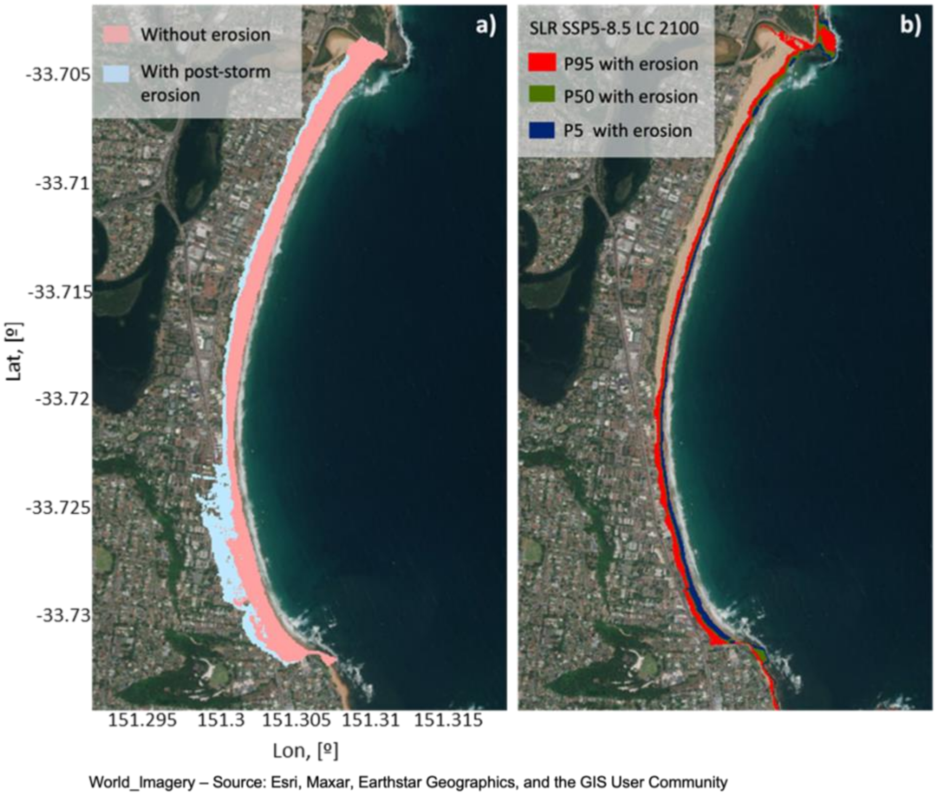

Figure S13. Storm and SLR-induced flooding extents with and without erosion. Flood extents with long-term and poststorm erosion (light blue) and without erosion (pink) for the 95% SLR percentile of the SSP5-8.5 for the low confidence scenario in 2100 (a). Permanent flooding for the SSP5-8.5 for the low confidence scenario in 2100 considering long-term erosion (b). Figure generated in ArcMap 10.8.1 using an Esri World Imagery base map (base map source: Esri, Maxar, Earthstar Geographics, and the GIS User Community).

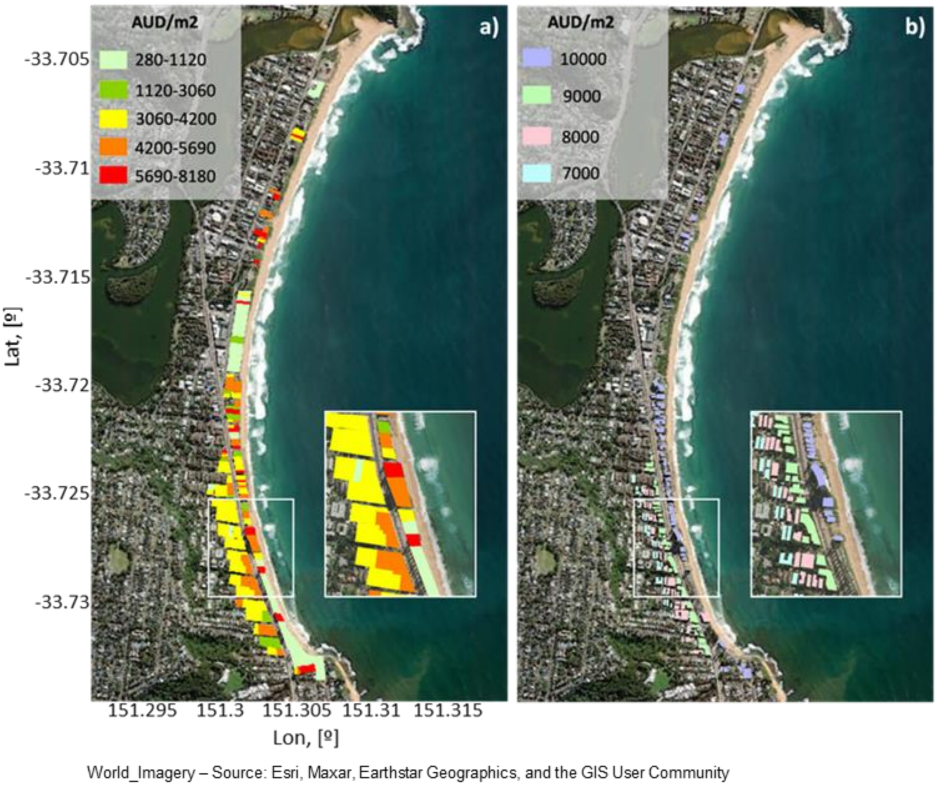

Figure S14. Land parcel value and building value. Land parcel value per square meter (a) and building value per square meter (b). Figure generated in ArcMap 10.8.1 using an Esri World Imagery base map (base map source: Esri, Maxar, Earthstar Geographics, and the GIS User Community).

Further details on the approaches considered

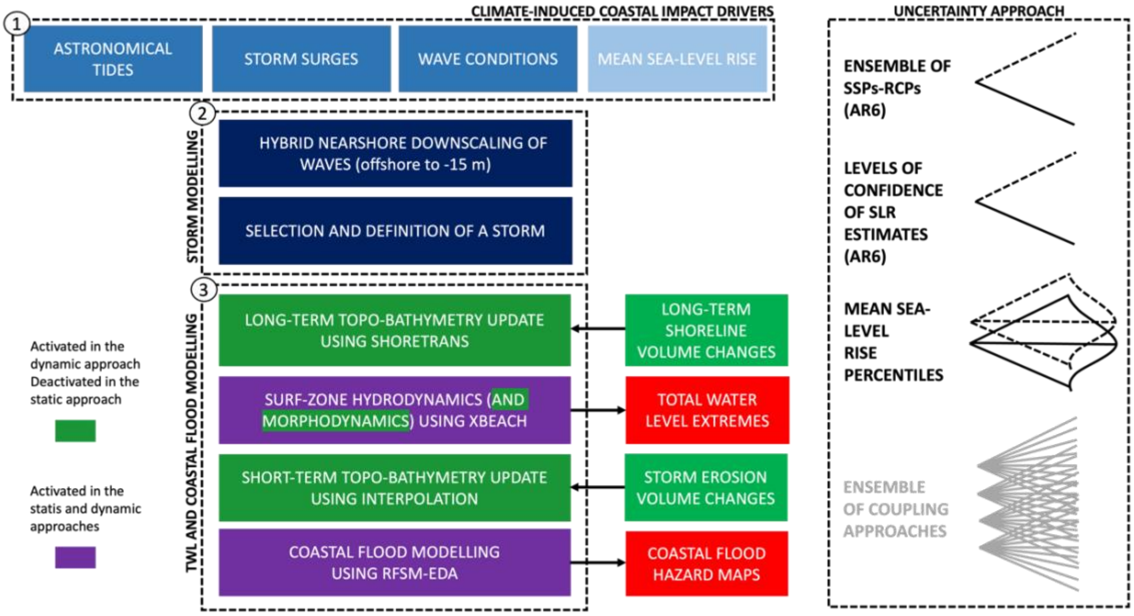

Figure S15. Flowchart of the methodology followed to implement the static and dynamic approaches presented in the main text. The panels in the left represent the three steps of the methodology from the impact drivers to the coastal flood modelling and the panel in the right highlights the uncertainty approach at each of the steps.

	**Dynamic approach**	**Static approach**	**Storm semi-dynamic approach**	**SLR semi-dynamic approach**
Processes considered in the TWL computation	Hydrodynamics and morphodynamics	Hydrodynamics	Hydrodynamics and morphodynamics	Hydrodynamics
Topo-bathymetries used in the hydraulic flood modelling	Long-term and short-term topo-bathymetry update	No topo-bathymetry update	Short-term topo-bathymetry update	Long-term topo-bathymetry update

Table S6 Summary of the main characteristics of the approaches considered in this study.

Glossary

**Term**	**Definition adopted in this article**
Beach flood protected area	Increase in flooded area resulting from flood modelling due to consideration of erosion compared to no consideration of erosion.
Beach flood protected value	The flood damage that occurs in the flood protection area. The flood protection value of the beach is therefore the benefit in terms of avoided flood damage if the present-day shoreline (or mean shoreline) were to remain fixed.
Beach nourishment	The adding of sediment onto or directly adjacent to an eroding beach ([25]).
Shoreline	Coastline including storm, seasonal and inter-annual variations (observed coastline).
Mean shoreline	Coastline excluding storm, seasonal and inter-annual variations (long-term coastline).
Resilience	The ability to bounce back and return to a previous state after a disturbance and the capacity for transformation ([26]).
Tipping points	Critical thresholds in a system that, when exceeded, can lead to a significant change in the state of the system, often with an understanding that the change is irreversible ([26]).

Table S7 Glossary with key terms and the definitions adopted in this article.

Supplementary References

1. Booij N, Ris RC, Holthuijsen LH (1999) A third-generation wave model for coastal regions 1. Model description and validation. *J. Geophys. Res. Oceans* 104, 7649–7666.
2. Camus P, Mendez FJ, Medina R, Cofiño AS (2011) Analysis of clustering and selection algorithms for the study of multivariate wave climate. *Coast. Eng.,* 58, 453-462.
3. Camus P, Mendez FJ, Medina R, Tomas A, Izaguirre C (2013) High-resolution downscaled ocean waves (DOW) reanalysis in coastal areas. *Coast. Eng.,* 72, 56-68.
4. McCarroll RJ, Masselink G, Valiente NG, Scott T, Wiggins M, Kirby J-A, Davidson M (2021) A rules-based shoreface translation and sediment budgeting tool for estimating coastal change: ShoreTrans. *Mar. Geol.,* 435, 106466.
5. Roelvink D, Reniers A, van Dongeren A, van Thiel de Vries J, McCall R, Lescinski J (2009) Modelling storm impacts on beaches, dunes, and barrier islands. *Coast Eng.,* 56: 1133-1152.
6. Jamieson S, L'homme J, Wright G, Gouldby B (2012) Highly efficient 2D inundation modelling with enhanced diffusion-wave and sub-element topography. *Proc. Inst. Wat. Man.,* 165(10): 581–595.
7. Handmer J, Reed C Percovich O (2002) Disaster Loss Assessment: Guidelines, Assessment. https://knowledge.aidr.org.au/media/1967/manual-27-disaster-loss-assessment-guidelines.pdf
8. Huizinga J, de Moel H, Szewczyk W (2017) Global flood depth-damage functions: Methodology and the database with guidelines, EUR 28552 EN, Publications Office of the European Union, Luxembourg, ISBN 978-92-79-67781-6, JRC105688.
9. Toimil A, Diaz-Simal P, Losada IJ, Camus P (2018) Estimating the risk of loss of beach recreation value under climate change. *Tour. Manag.*, 68, 387-400.
10. Fox-Kemper B, Hewitt HT, Xiao C et al. (2021) Ocean, Cryosphere and Sea Level Change. In Climate Change 2021: The Physical Science Basis. Contribution of Working Group I to the Sixth Assessment Report of the Intergovernmental Panel on Climate Change [Masson-Delmotte V et al. (eds.)]. Cambridge University Press, Cambridge, UK and New York, NY, USA, pp. 1211–1362.
11. Lobeto H, Menendez M, Losada IJ (2021) Future behavior of wind wave extremes due to climate change. *Sci. Rep.*, 11(1), 1-12.
12. Tebaldi C, Ranasinghe R, Vousdoukas M, Rasmussen DJ, Vega-Westhoff B, Kirezci E., ... & Mentaschi L (2021) Extreme sea-levels at different global warming levels. *Nat. Clim. Change*, 1-6
13. Alvarez-Cuesta M, Toimil A, Losada IJ (2021) Modelling long-term shoreline evolution in highly anthropized coastal areas. Part 1: Model description and validation. *Coast. Eng*., 169, 103960.
14. Matheen N, Harley MD, Turner IL, Splinter KD, Simmons JA, Thran MC (2021) Bathymetric data requirement for operational coastal erosion forecasting using XBeach. *J. Mar. Sci. Eng.*, 9(10), 1053.
15. McEvoy S, Haasnoot M, Biesbroek R (2021) How are European countries planning for sea level rise? *Ocean Coast Manag.*, 203, 105512.
16. Stammer D, Van de Wal RSW, Nicholls RJ, Church JA, Le Cozannet G et al. (2019) Framework for high-end estimates of sea level rise for stakeholder applications. *Earth's Future*, 7(8), 923-938.
17. Nicholls RJ, Hanson SE, Lowe JA, Warrick RA, Lu X, Long AJ (2014) Sea-level scenarios for evaluating coastal impacts. *Wiley Interdiscip. Rev. Clim. Change,* 5(1), 129-150.
18. Hinkel J, Church JA, Gregory JM, Lambert E, Le Cozannet G et al. (2019) Meeting user needs for sea level rise information: a decision analysis perspective. *Earth's Future*, 7(3), 320-337.
19. Durand G, van den Broeke MR, Le Cozannet G, Edwards TL, Holland PR, Jourdain NC, Marzeion B, Mottram R, Nicholls RJ, Pattyn F, Paul F, Slangen ABA et al. (2022) Sea-Level Rise: From Global Perspectives to Local Services. *Front. Mar. Sci.*, 8, 709595.
20. Edwards TL, Nowicki S, Marzeion B, Hock R, Goelzer H, Seroussi H, ... & Zwinger T (2021) Projected land ice contributions to twenty-first-century sea level rise. *Nature*, 593(7857), 74-82.
21. Bamber JL, Oppenheimer M, Kopp RE, Aspinall WP, Cooke RM (2019) Ice sheet contributions to future sea-level rise from structured expert judgment. *PNAS*, 116(23), 11195-11200.
22. Levermann A, Winkelmann R, Albrecht T, Goelzer H, Golledge NR, Greve R, ... & Van De Wal RS (2020) Projecting Antarctica's contribution to future sea level rise from basal ice shelf melt using linear response functions of 16 ice sheet models (LARMIP-2). *Earth Syst Dyn.*, 11(1), 35-76.
23. DeConto RM, Pollard D, Alley RB, Velicogna I, Gasson E, Gomez N, ... & Dutton A (2021) The Paris Climate Agreement and future sea-level rise from Antarctica. *Nature*, 593(7857), 83-89.
24. Turner IL, Harley MD, Short AD, Simmons JA, Bracs MA, Phillips MS, Splinter KD (2016) A multi-decade dataset of monthly beach profile surveys and inshore wave forcing at Narrabeen, Australia. *Sci. Data*, 3(1), 1-13.
25. USACE (1956) Link: https://www.iwr.usace.army.mil/Missions/Coasts/Tales-of-the-Coast/Corps-and-the-Coast/Shore-Protection/Beach-Nourishment/ (last accessed January 2023).

26. IPCC (2022) Climate Change 2022: Impacts, Adaptation and Vulnerability. Contribution of Working Group II to the Sixth Assessment Report of the Intergovernmental Panel on Climate Change [H-O Pörtner et al. (eds.)]. Cambridge University Press. Cambridge University Press, Cambridge, UK and New York, NY, USA, 3056 pp.
